# Identification of polyketide synthase genes required for aspinolide biosynthesis in *Trichoderma arundinaceum*

**DOI:** 10.1007/s00253-022-12182-9

**Published:** 2022-09-27

**Authors:** Rosa E. Cardoza, Susan P. McCormick, Inmaculada Izquierdo-Bueno, Natalia Martínez-Reyes, Laura Lindo, Daren W. Brown, Isidro G. Collado, Robert H. Proctor, Santiago Gutiérrez

**Affiliations:** 1 University Group for Research in Engineering and Sustainable Agriculture (GUIIAS), Area of Microbiology, University of León, 24400 Ponferrada, Spain; 2 USDA, Agricultural Research Service, National Center for Agricultural Utilization Research, Mycotoxin Prevention and Applied Microbiology Research Unit, 1815 N University St., Peoria, IL 61604 USA; 3 Departamento de Química Orgánica, Facultad de Ciencias, Universidad de Cádiz, Campus Universitario Río San Pedro s/n, Torre Sur, 4ª planta, 11510 Puerto Real, Cádiz Spain

**Keywords:** Aspinolides, *Trichoderma*, Trichothecenes, Metabolomics, Gene clusters, Comparative genomics

## Abstract

**Abstract:**

The fungus *Trichoderma arundinaceum* exhibits biological control activity against crop diseases caused by other fungi. Two mechanisms that likely contribute to this activity are upregulation of plant defenses and production of two types of antifungal secondary metabolites: the sesquiterpenoid harzianum A (HA) and the polyketide-derived aspinolides. The goal of the current study was to identify aspinolide biosynthetic genes as part of an effort to understand how these metabolites contribute to the biological control activity of *T. arundinaceum*. Comparative genomics identified two polyketide synthase genes (*asp1* and *asp2*) that occur in *T. arundinaceum* and *Aspergillus ochraceus*, which also produces aspinolides. Gene deletion and biochemical analyses in *T. arundinaceum* indicated that both genes are required for aspinolide production: *asp2* for formation of a 10-member lactone ring and *asp1* for formation of a butenoyl subsituent at position 8 of the lactone ring. Gene expression and comparative genomics analyses indicated that *asp1* and *asp2* are located within a gene cluster that occurs in both *T. arundinaceum* and *A. ochraceus*. A survey of genome sequences representing 35 phylogenetically diverse *Trichoderma* species revealed that intact homologs of the cluster occurred in only two other species, which also produced aspinolides. An *asp2* mutant inhibited fungal growth more than the wild type, but an *asp1* mutant did not, and the greater inhibition by the *asp2* mutant coincided with increased HA production. These findings indicate that *asp1* and *asp2* are aspinolide biosynthetic genes and that loss of either aspinolide or HA production in *T. arundinaceum* can be accompanied by increased production of the other metabolite(s).

**Key points:**

• *Two polyketide synthase genes are required for aspinolide biosynthesis.*

• *Blocking aspinolide production increases production of the terpenoid harzianum A.*

• *Aspinolides and harzianum A act redundantly in antibiosis of T. arundinaceum.*

**Supplementary Information:**

The online version contains supplementary material available at 10.1007/s00253-022-12182-9.

## Introduction

Multiple species of the fungus *Trichoderma* are used for biological control of plant pathogenic fungi, and secretion of antimicrobial secondary metabolites (antibiosis) contributes to the biological control (Cardoza et al. 2011; Degenkolb et al. 2008; Hermosa et al. 2014; Nielsen et al. 2005; Sivasithamparam and Ghisalberti 1998). For example, the ability of *Trichoderma arundinaceum* to limit disease caused by the plant pathogenic fungi *Botrytis cinerea* and *Rhizoctonia solani* has been attributed, in part, to production of harzianum A (HA), a secondary metabolite (SM) that is a member of the trichothecence family of toxic sesquiterpenoids (Malmierca et al. 2012, 2013). However, HA-nonproducing mutants of the fungus retain varying but significant levels of antifungal activity. Retention of the activity has been attributed to the production of aspinolides, a family of antimicrobial polyketide-derived SMs that are produced at higher levels in HA-nonproducing mutants than in wild-type *T. arundinaceum* (Lindo et al. 2018, 2019a, 2019b; Malmierca et al. 2015, 2013, 2012).

Although recent studies report aspinolide production by *T. arundinaceum* (phylum Ascomycota, class Sordariomycetes), production was first reported in the relatively distantly related fungus *Aspergillus ochraceus* (phylum Ascomycota, class Eurotiomycetes) (Fuchser and Zeeck 1997). This first report included precursor feeding experiments with labeled acetate and O_2_ gas. From these experiments and the deduced structure of several aspinolide analogs, the authors proposed an aspinolide biosynthetic pathway in which both the aspinolide backbone, a 10-member lactone ring, and a butenoyl substituent are polyketides (Fuchser and Zeeck 1997).

**Fig. 1:**
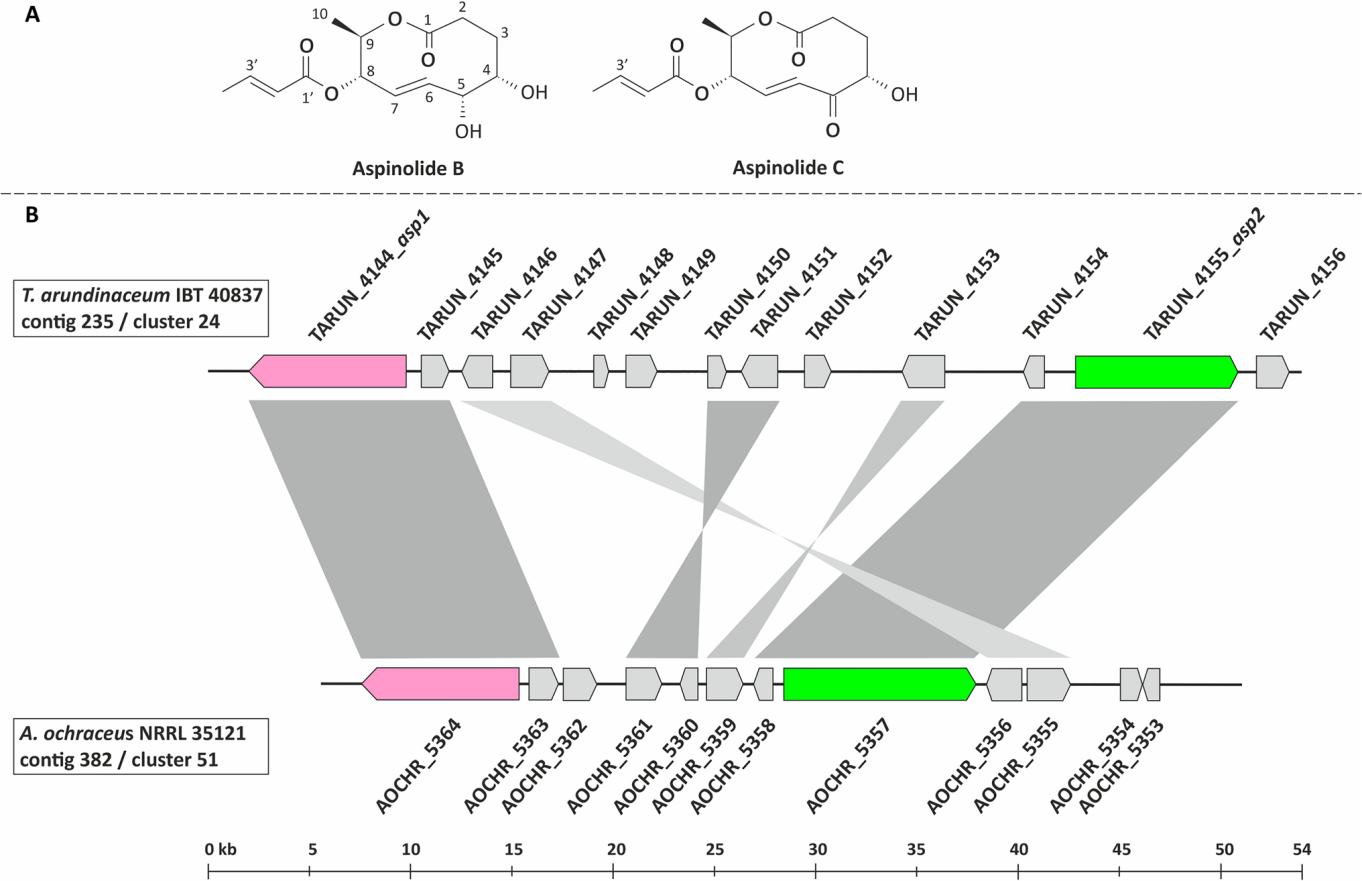
**A** Chemical structure of aspinolides B and C, the two major aspinolides produced by *Trichoderma arundinaceum* (Malmierca et al. 2015; Izquierdo-Bueno et al. 2018). **B** Comparison of the putative aspinolide biosynthetic gene clusters in *T. arundinaceum* IBT 40837 and *A. ochraceus* NRRL 35121. The predicted functions of the genes included in the clusters in *T. arundinaceum* and *A. ochraceus* are indicated in Table 1 and Table S3, respectively. Gray shading indicates gene synteny shared by the two clusters. The pink and green arrows indicate the proposed genes, *asp1* and *asp2*, respectively.

Nine aspinolide analogs (AspB-AspJ) have been identified in cultures of wild-type and HA-nonproducing strains of *T. arundinaceum*, but not all strains produced all analogs (Fig. 1A, Figure S1) (Izquierdo-Bueno et al. 2018; Malmierca et al. 2015). Aspinolide production by *T. arundinaceum* is dependent on culture conditions and is generally inversely proportional to HA production. Although only low levels of AspB and AspC are produced at early time points in cultures of wild-type *T. arundinaceum*, both are produced at markedly higher levels in cultures of HA-nonproducing mutants (Izquierdo-Bueno et al. 2018; Lindo et al. 2018). Interestingly, the biological activity of AspC is similar to that of HA in that both metabolites exhibit antifungal activity against *B. cinerea* and in vitro phytotoxicity against tomato. In contrast, some other aspinolide analogs (e.g., AspB and AspF) lack these activities (Malmierca et al. 2015). In addition, both HA and AspC can upregulate the expression of plant defense-related genes in tomato (Malmierca et al. 2015).

There is little information available on the genetics of aspinolide biosynthesis in *T. arundinaceum*. By contrast, there is substantial information on the genetics of HA biosynthesis, and this information has provided important insights into the role of HA in the ecology of the fungus (Gutiérrez et al. 2021; Proctor et al. 2020). In *T. arundinaceum*, trichothecene biosynthetic genes (*tri* genes) occur at three loci: the large *tri* cluster, small *tri* cluster, and *tri5* locus. Most of the genes at these loci have closely related homologs in other trichothecene-producing species of *Trichoderma* and/or other fungal genera, such as *Fusarium*, *Myrothecium*, and *Trichothecium* (Gutiérrez et al. 2021; Proctor et al. 2020). The chemical structure common to all trichothecene analogs (12,13-epoxytrichothec-9-ene, EPT) consists of two 6-member rings, one 5-member ring, and an epoxide group. The *tri5*-encoded terpene synthase catalyzes the conversion of the primary metabolite farnesyl diphosphate (FDP) to trichodiene, the parent compound of all trichothecene analogs. Enzymes encoded by three genes in the large *tri* cluster catalyze the conversion of trichodiene to trichodermin (4-acetyl EPT), and enzymes encoded by the three genes in the small *tri* cluster catalyze the conversion of trichodermin to HA (4-octatrienedioyl EPT).

The objective of the current study was to identify aspinolide biosynthetic genes in order to investigate the role of aspinolides in the biocontrol activity of *T. arundinaceum*. To accomplish this goal, we used a three-step approach. First, we identified an aspinolide-producing strain of *Aspergillus*; second, we used comparative genomics to identify closely related polyketide synthase (PKS) gene homologs in *T. arundinaceum* and an aspinolide-producing *Aspergillus* strain; and third, we used gene deletion and biochemical analyses to determine whether the PKS genes are required for aspinolide production. From this approach, we identified two PKS genes as well as a putative aspinolide biosynthetic gene cluster in both *T. arundinaceum* and *A. ochraceus*. We then used mutant strains of *T. arundinaceum* in which the PKS genes were deleted to examine how aspinolides impact antifungal activity of *T. arundinaceum*. Finally, we proposed an updated aspinolide biosynthetic pathway. Together, the results of this study provide a basis for further investigations into the complex nature of antifungal activity of *T. arundinaceum*.

## Material and methods

### Strains used and culture conditions

*Trichoderma arundinaceum* IBT 40837 (= CBS 119573 = Ta37) was used as the wild-type progenitor strain for the gene deletion studies and for the genomic, metabolomic, and transcriptomic analyses. *Tylophoron protrudens* CBS 121320, *T. rodmanii* CBS 121553, and *T. turrialbense* CBS 112445, also members of the Brevicompactum clade, were used to assess the production of aspinolides in species other that *T. arundinaceum*. *Trichoderma* strains were maintained on potato-dextrose agar medium (PDA), which was prepared from PDB broth (Becton Dickinson Co., Sparks, MD) amended with 2.5% agar (Oxoid, Ltd.). Sporulation occurred during growth on PDA at 28 °C in the dark for 7 days.

Aspinolide production was assessed in three strains of *Aspergillus ochraceus*: NRRL 35158, NRRL 35121, and NRRL 62072, and six strains of *Aspergillus westerdijkiae*: NRRL 35061, NRRL 35072, NRRL 3174, NRRL 6160, NRRL 35460, and NRRL 35050 obtained from the ARS Culture Collection (U.S. Department of Agriculture, Agriculture Research Service). *Aspergillus* strains were grown, and sporulation was induced under the conditions described above for *Trichoderma* except that the incubation temperature was 30 °C.

The filamentous fungus *Rhizoctonia solani* strain R43 and the yeast *Kluyveromyces marxianus* strain CECT 1018 were used in antifungal assays. *Rhizoctonia solani* was grown on PDA medium at 28 °C for 5–7 days. *Kluyveromyces marxianus* CECT 1018 was grown on malt extract agar (MEA) medium (2% malt extract, 0.1% peptone, 2% glucose, 2% agar) at 30 °C for 2–3 days.

### Constructions of gene deletion plasmids pΔ*asp1* and pΔ*asp2*

For the construction of *asp1* deletion plasmid pΔ*asp1*, DNA regions adjacent to *T. arundinaceum asp1* were PCR amplified using oligonucleotides Asp1-5r_F_*Bam*HI/Asp1_5r_*Sma*I and Asp1_3r_F_*Sma*I/ Asp1_3r_R_*Xho*I (Table S1). The fragments 5′ (1,117 bp) and 3′ (1,218 bp) were digested with *Bam*HI-*Sma*I and *Sma*I-*Xho*I, respectively, and after gel purification were ligated into pBluescript KS + (Stratagene, La Jolla, CA) previously digested with *Bam*HI and *Xho*I. The resulting plasmid, pBasp1-5r3r (5,252 bp) was linearized with *Sma*I, dephosphorylated with alkaline phosphatase (Thermo Scientific), and ligated to a 2,710 bp fragment containing the *hyg* resistance cassette, which was isolated from pAN7-1 (Punt et al. 1987) by digestion with *Ecl*136II-*Hin*dIII, and treated with Klenow fragment (Thermo Scientific, Waltham, MA), to finally obtain plasmid pΔ*asp1* (7,960 bp) (Figure S2A).

Similarly, for construction of *asp2* deletion plasmid pΔ*asp2*, DNA regions adjacent to *T. arundinaceum asp2* were PCR amplified using primer pairs Asp2_5r_F_*Xba*I/Asp1_5r_R_*Sma*I and Asp1_3r_F_*Sma*I/Asp1_3r_R_*Xho*I (Table S1). The fragments 5′ (1227 bp) and 3′ (1142 bp) were digested with *Xba*I-*Sma*I and *Sma*I-*Xho*I, respectively, and after gel purification were ligated into pBluescript KS + vector (Stratagene) previously digested with *Xba*I and *Xho*I, creating pBasp2-5r3r (5,279 bp). Similar to above, the *hyg* resistance cassette was then cloned into pBasp2-5r3r creating pΔ*asp2* (7,985 bp) (Figure S2B).

### Trichoderma arundinaceum transformation and selection of transformants

Ten micrograms of either plasmid pΔ*asp1* or pΔ*asp2*, linearized with *Xho*I, were transformed into protoplasts of *T. arundinaceum* Ta37. Putative transformants were selected for resistance to hygromycin (250 μg/mL), as previously described (Cardoza et al. 2006; Proctor et al. 1999).

### Metabolomics characterization and antifungal activity

#### Quantification of HA by HPLC and determination of the antifungal activity on plate assays (antibiogram)

To assess HA production, a 500-mL flask containing 100 mL of malt complex (CM) medium (0.5% malt extract, 0.5% yeast extract, 0.5% glucose) was inoculated with 2 × 10^6^ spores/mL and incubated for 48 h at 28 °C and 250 rpm in an orbital shaker. Twenty milliliters of this pre-culture was then transferred to a new 500-mL flask containing 100 mL of potato-dextrose broth (PDB) medium (Difco, Becton Dickinson) as described previously (Cardoza et al. 2011) and incubated in an orbital shaker as described above. After 48 h, mycelia were harvested by filtration through sterile Nytal filters (Sefar Maissa S.A., Barcelona, Spain), and the resulting culture filtrates stored at – 80 °C until metabolomics analysis. HA was purified and quantified by HPLC from liquid PDB cultures of *T. arundinaceum* strains as previously described (Cardoza et al. 2011).

Antibiograms against *Kluyveromyces marxianus* CECT 1018 were carried out as previously described (Cardoza et al. 2011), and antifungal assays against *Rhizoctonia solani* R43 on cellophane membranes were performed as described by Gutiérrez et al. (2021).

#### Gas chromatography-mass spectrometry (GC-MS) analysis for aspinolide detection

To assess aspinolide production, 20 mL of YEPD (0.1% yeast extract, 0.1% peptone, 2% glucose) medium in a 50-mL Erlenmeyer flask were inoculated with spores from one plate of V8 agar medium (Difco, Becton Dickinson) and incubated at 28 °C, 200 rpm. After 7 days of growth, cultures were extracted with 8 mL ethyl acetate. Extracts were dried under a stream of nitrogen and the residue was resuspended in 1 mL of ethyl acetate. GC-MS analyses of the extracts were performed on a Hewlett Packard 6890 gas chromatograph fitted with a HP-5MS column (30 m, 0.25 mm, 0.25 μm) and a 5973-mass detector. The carrier gas was helium with a 20:1 split ratio and a 20 mL/min split flow. The column was held at 150 °C for 1 min following injection, heated to 280 °C at 30 °C/min, and held for 7.7 min. Compound identifications were based on GC-MS comparisons with purified standards of aspinolides B and C. In the GC-MS analysis, the presence of aspinolide analogs was detected using a fragment ion with m/z 69, which is derived from the 8-butenoyl substituent of aspinolides (Malmierca et al. 2015).

#### Isolation of aspinolides and NMR analysis

To generate sufficient quantity of aspinolides for ^1^H-nuclear magnetic resonance (NMR) analysis, 1-cm diameter mycelia plugs, collected from 5 days old PDA plates, were used to inoculate 40 Petri dishes (150-mm diameter) containing 100 mL MEA and incubated at 25 °C in presence of white artificial light for 12 days. The mycelium was scraped from the agar surface and discarded. The agar medium was then fragmented and extracted with ethyl acetate in 500-mL flasks in an ultrasonic bath for 15 min. The extract was filtered to remove the agar debris and dried using a rotary evaporator at low pressure.

The extracts were fractionated by silica gel column chromatography (CC), eluting with mixtures containing increasing percentages of ethyl acetate/hexane (10–100%) and methanol as the mobile phase. TLC and extensive spectroscopic analyses by ^1^H NMR and ^13^C NMR were used to detect the presence of the various metabolites in each fraction. Final purification of metabolites from selected fractions was carried out by HPLC. Metabolites were eluted with a gradient of ethyl acetate in hexane (10–100%) at a flow rate of 1 mL/min. The column was then rinsed with 100% methanol.

Purification by semipreparative and analytical HPLC was performed with a Hitachi/Merck L-7100 apparatus equipped with a differential refractometer detector (RI-7490) and either a LiChrospher® Si 60 (5 μm) LiChroCart® (250 mm × 4 mm) or a LiChrospher® Si 60 (10 μm) LiChroCart® (250 mm × 10 mm) column. Silica gel (Merk) was used for column chromatography. TLC was performed using a 0.25-mm-thick Merk Kieselgel 60 F_254_ plate.

^1^H and ^13^C NMR measurements were recorded on Agilent 400 MHz and Agilent 500 MHz spectrometers with SiMe_4_ as the internal reference. Chemical shifts were referenced to CDCl_3_ (δ_H_ 7.25, δ_C_ 77.0). NMR assignments were made using a combination of 1D and 2D techniques. Multiplicities are described using the following abbreviations: s, singlet; d, doublet; t, triplet; q, quarter; quint, quintuplet; sext, sextuplet; m, multiplet; br; broad. High-resolution mass spectroscopy (HRMS) was recorded with a QTOF mass spectrometer in positive ion electrospray mode with a cone voltage of 20 V.

### Quantification of ergosterol and squalene

Total intracellular sterols were extracted from the mycelia harvested from the same cultures used for HA quantification. Ergosterol and squalene contents were calculated as reported previously (Cardoza et al. 2007; Ghimire et al. 2009).

### Genome sequence of A. ochraceus NRRL 35121 and Δasp1.9 and Δasp2.3 mutants and bioinformatic analysis

Genome sequences were obtained with an Illumina MiSeq platform (Illumina, Inc.). To prepare DNA for genome sequencing, *A. ochraceus* NRRL 35121 wild-type strain, and Δ*asp1* and Δ*asp2* mutant strains were grown in YEPD medium for 7 days, at room temperature with shaking at 200 rpm. Mycelia were harvested by filtration, lyophilized, ground to a powder, and genomic DNA was extracted using the ZR Fungal/Bacterial DNA MiniPrep kit (Zymo Research). Libraries for DNA sequencing were prepared using the Nextera XT DNA library Preparation Kit as specified by the manufacturer (Illumina). Sequence reads were processed and assembled using CLC Genomics Workbench (Qiagen Inc.). The resulting assembly of *A. ochraceus* NRRL 35121 genomic sequence was submitted to the GenBank/National Center for Biotechnology Information (NCBI) database as accession JANYMM000000000.

### RNAseq analysis

RNA was extracted from mycelia of wild-type *T. arundinaceum* and the *tri6* mutant strain Δtri6.66 grown for 48 h in PDB medium as previously described (Cardoza et al. 2015). cDNA synthesis, library construction, sequencing, and analysis were carried out as described previously (Lindo et al. 2018). Briefly, relative transcription levels for each gene model were reported as TPM (transcripts per million) (Li et al. 2010), which was computed as RPKMi × 10^6^/∑j RPKMj, where RPKM (reads per kilobase per million mapped reads) (Mortazavi et al. 2008) corresponds to the total exon reads/mapped reads (millions) for a particular gene (i) or for all the *T. arundinaceum* genes (j). A total of 8,396,204 trimmed reads (average length = avl = 122.8 bp) were obtained for the wild-type samples, and 11,900,790 (avl = 127.3 bp) trimmed reads were obtained for the Δ*tri6* mutant. Where noted, RNAseq data corresponding to accession SRP156794 were retrieved from the GenBank Sequence Read Archive (SRA) database (Lindo et al. 2018).

### Antifungal assays

Antibiograms against *Kluyveromyces marxianus* CECT 1018 (Cardoza et al. 2011) and antifungal assays on membranes against *Rhizoctonia solani* R43 (Gutiérrez et al. 2021) were carried out as described previously. The degree of radial growth inhibition of *R. solani* was calculated as previously described (Cardoza et al. 2007; Royse and Ries 1978).

### Phylogenetic analysis

The full-length predicted amino acid sequences of ASP1, ASP2, and 159 fungal PKSs with known functions as well as the fatty acid synthase (FAS) from *Gallus gallus*, which was added as an outgroup (Brown et al. 2022), were aligned using Muscle as implemented in MEGA X (Kumar et al. 2018). The evolutionary history of the PKSs was then inferred using the Neighbor-Joining and Maximum Parsimony methods in MEGA X (Nei and Kumar 2000). The percentage of trees in which the associated taxa clustered together in the bootstrap test (500 replicates) is shown next to the branches.

Homologs of *asp1* and *asp2* in other fungi were identified by BLASTx analysis in which the DNA sequence of the *T. arundinaceum* genes TARUN_4144 and TARUN_4155 were used as queries against the GenBank/NCBI nonredundant fungal database. The BLASTx analysis using the *asp1* and *asp2* as queries was then repeated using databases that were limited to the fungal species corresponding to the 10 best hits for each query in the initial analysis. This facilitated identification of fungi that had homologs of both *asp1* and *asp2*. Genome sequence data in the NCBI/GenBank database were used to determine whether the *asp1* and *asp2* homologs were located near one another and, therefore, potentially in the same gene cluster. The predicted amino acid sequences of the *asp1* and *asp2* homologs identified from the BLASTx analysis were aligned with sequences from *A. ochraceus* and *Trichoderma* homologs using Muscle, as described above. The resulting alignments were then subjected to maximum likelihood analysis with ultrafast bootstrapping as implemented in the program IQ-Tree version 1.6.7 (Minh et al. 2013; Nguyen et al. 2015).

## Results

### Comparative analyses to identify candidate aspinolide biosynthetic PKS genes

#### Rationale for aspinolide PKS gene identification strategy

The 10-member lactone ring of aspinolides and the butenoate substituent esterified at carbon atom 8 (C-8) of the lactone ring have a pentaketide and diketide origin, respectively (Fuchser and Zeeck 1997; Malmierca et al. 2015). Based on biochemical considerations and comparison to lovastatin biosynthesis, which requires a nonaketide and a diketide synthesized by two different PKS enzymes, the pentaketide and diketide precursors of aspinolide biosynthesis are likely synthesized by two different PKS enzymes (Fig. 1A).

Species of *Aspergillus* and *Trichoderma* are reported to produce aspinolides. Although these genera are both in the phylum Ascomycota, they are in different classes (Eurotiomycetes and Sordariomycetes) and, therefore, distantly related. Given this distant relationship, SM biosynthetic genes in the two genera should also be, in general, distantly related. However, if aspinolide biosynthesis requires two PKS genes, aspinolide-producing species of *Aspergillus* and *Trichoderma* should have closely related homologs of the two PKS genes. Furthermore, because enzyme-encoding genes involved in synthesis of the same SM tend to be clustered in fungi, the two putative aspinolide PKS genes should be located near one another in the genome sequences of the aspinolide-producing *Aspergillus* and *Trichoderma* species.

#### Aspinolide-producing strains

We selected *T. arundinaceum* strain IBT 40837 for this study based on its availability and its demonstrated ability to produce aspinolides (Malmierca et al. 2015). Because the *A. ochraceus* strain with a demonstrated ability to produce aspinolides was not available, we examined aspinolide production in multiple strains of both *A. ochraceus* and *A. westerdijkiae*. The strains of *A. westerdijkiae* were included because *A. ochraceus* was recently resolved into two species, *A. ochraceus* (sensu stricto), and *A. westerdijkiae* (Frisvad et al. 2014), and it is not clear which of these two species was previously examined for aspinolide production (Fuchser and Zeeck 1997). Of the nine *Aspergillus* strains examined, aspinolide production was detected in one *A. ochraceus* strain (NRRL 35121) and two *A. westerdijkiae* strains (NRRL 35050 and NRRL 35072). Only one aspinolide analog (aspinolide B) was detected in the three strains. Based on these results, *A. ochraceus* NRRL 35121 was selected for further analysis.

#### Comparison of predicted PKS genes in *A. ochraceus* and *T. arundinaceum*

To identify the two putative aspinolide PKS genes, we used tBLASTn analysis in which the genome sequence of *A. ochraceus* NRRL 35121 was queried with the amino acid sequences of the 23 predicted PKSs from *T. arundinaceum*. In the tBLASTn results, the Expect-values (*E*-values) for most of the hits in the *A. ochraceus* genome sequence were between 1.25 × 10^−7^ and 3.30 × 10^−79^. However, a hit on *A. ochraceus* contig 382 to the *T. arundinaceum* PKS protein associated with locus tag TARUN_4144 had an *E*-value of 0.00. Contig 382 also contained a second hit, located ~ 12 Kb from the first hit, with an *E*-value of 1.07 × 10^−33^ to the *T. arundinaceum* PKS protein TARUN_4155. In *T. arundinaceum*, PKS genes TARUN_4144 and TARUN_4155 were located 33 kb apart on the same contig (Table S2). To help determine whether the two PKS genes could be part of the same SM biosynthetic gene cluster, we examined the results of antiSMASH analysis of the *A. ochraceus* and *T. arundinaceum* genome sequences. In both fungi, the two PKS genes were in one predicted SM biosynthetic gene cluster (Fig. 1B, Table 1, Table S3). The putative aspinolide clusters in the two fungi also include closely related homologs of seven other genes, and the relative organization of these nine genes was partially conserved (Fig. 1B). Hereafter, the two PKS genes are referred to as *asp1* (= TARUN_4144 and AOCHR_5364) and *asp2* (= TARUN_4155 and AOCHR_5357).

**Table 1:** Genes predicted in the genomic region of *T. arundinaceum* containing *asp1* and *asp2* genes

Gene	Protein (aa)	Predicted function Highest identity	Blastp *E*-value; Accession No
TARUN_4144 (*asp1)*	2521	Diketide synthase (PKS)	0.0; KXX76480.1
TARUN_4145	500	Acetyl/acyl transferase	0.0; KXX76481.1
TARUN_4146	422	Cytochrome P450	0.0; KXX76482.1
TARUN_4147	612	Cytochrome P450	0.0; KXX76483.1
TARUN_4148	240	Unknown	3e^−152^; PKK53437.1
TARUN_4149	506	FAD-dependent monooxygenase	0.0; KXX76484.1
TARUN_4150	236	Short-chain dehydrogenase-reductase	1e^−126^; PKK53440.1
TARUN_4151	586	FAD-linked oxidoreductase	0.0; KXX80981
TARUN_4152	349	Unknown	2e^−175^; OPB46113.1
TARUN_4153	513	Cytochrome P450	8e^−150^; XP_022490940
TARUN_4154	306	Alpha/beta-hydrolase	1e^−122^; PMD39540.1
TARUN_4155 (*asp2*)	2338	Pentaketide synthase	0.0; XP_007584097.1
TARUN_4156	520	Zn2Cys6 transcription factor	0.0; OPB46110.1

### Deletion of *asp1* and *asp2* genes

#### *asp1* gene disruption

Transformation of the wild-type *T. arundinaceum* strain with deletion plasmid pΔ*asp1* (Figure S2A) yielded 67 hygromycin-resistant transformants. Initial PCR analyses yielded 15 transformants with amplicon patterns consistent with deletion of *asp1*. That is, a 466-bp amplicon with the primer pair (Db741 and Db742) designed to amplify an internal 466-bp *hph* fragment and no amplicon with the primer pair (Asp1R and Asp1F) designed to amplify an internal 1,028-bp *asp1* fragment. Subsequent PCR analyses yielded one transformant, Δasp1.9, with amplicon patterns consistent with deletion of the *asp1* coding region via integration of the *asp1* deletion cassette from pΔ*asp1* into the target region by double homologous recombination. That is, a 1,491-bp amplicon with the primer pair (Asp1_5rr and TtrpC-d) designed to detect integration of the deletion cassette into the 5’ flanking region of *asp1* and a 1,410-bp amplicon with the primer pair (Asp1_3rr and PgpdA_d) designed to detect integration of the deletion cassette into the 3’ flanking region of *asp1* (Figure S3A, Figure S4). Sanger sequence analysis of the latter two amplicons provided additional evidence that the recombination process occurred as designed. Genome sequence analysis confirmed deletion of *asp1* in transformant Δasp1.9 (Figure S3B).

#### *asp2* gene deletion

Transformation of wild-type *T. arundinaceum* with deletion plasmid pΔ*asp2* (Figure S2B) yielded 35 hygromycin-resistant transformants. PCR analysis yielded four transformants with amplicon patterns consistent with the deletion of the *asp2* coding region via integration of the *asp2* deletion cassette by double homologous recombination. That is, the transformants yielded a 1,603-bp amplicon with the primer pair (Asp2-5rr and TtrpC-d) designed to detect integration of the deletion cassette in the 5’ flanking region of *asp2* and a 1,338-bp amplicon with the primer pair (PgpdA-d and Asp2-3rr) designed to detect integration of the cassette in the 3’ flanking region of *asp2* (Table S1) (Figure S5A, Figure S6). Sanger sequence analysis of the latter amplicons from two of the transformants (Δasp2.3 and Δasp2.18) provided additional evidence that the recombination process occurred as designed. Transformant Δasp2.3 was selected for further analyses. Genome sequence analysis which confirmed deletion of *asp2* in transformant Δasp2.3 (Figure S5B).

### Targeted metabolomics of Δ*asp1*- and Δ*asp2* mutants

#### Lack of aspinolide GC–MS signals in Δ*asp1* mutant

Initial GC-MS analysis, focused on *m/z (mass-to-charge)* 69 ion specific for detection of aspinolides, was carried out with Δ*asp1* mutant and the wild-type strain. Four aspinolide analogs were detected in cultures of the wild-type strain, but none of the analogs was detected in cultures of the Δ*asp1* mutant. Thus, *asp1* deletion abolished the wild-type aspinolide production phenotype (Figure S7).

#### Production of known aspinolide analogs

To further examine aspinolide production, large-scale chromatographic separations of extracts from cultures of Δ*asp1*, Δ*asp2*, and wild-type strains were conducted. Aspinolides B and C were present at 24.4 and 14.3 mg/L, respectively, in extracts of the wild-type strain but were not detected in extracts of the Δ*asp1* and Δ*asp2* mutants (Table 2).

**Table 2:** Production of metabolites (mg L^−1^) by Ta37, Δ*asp1*, and Δ*asp2* (12 days post-inoculation)

Strain	Total extract	Fatty acids	AspB	AspC	Compound **1** 8-Hydroxy aspinolide A	Compound **2** 3,11-Diepiisotrichotriol	HA
Ta37	317.3	152.5	24.4	14.3	-	-	40.3
Δasp1.9	781.5	407.5	-	-	2.3	3.9	35.3
Δasp2.3	635.1	358.3	-	-	-	3.6	41.8

#### Production of novel aspinolide analog

**Fig. 2:**
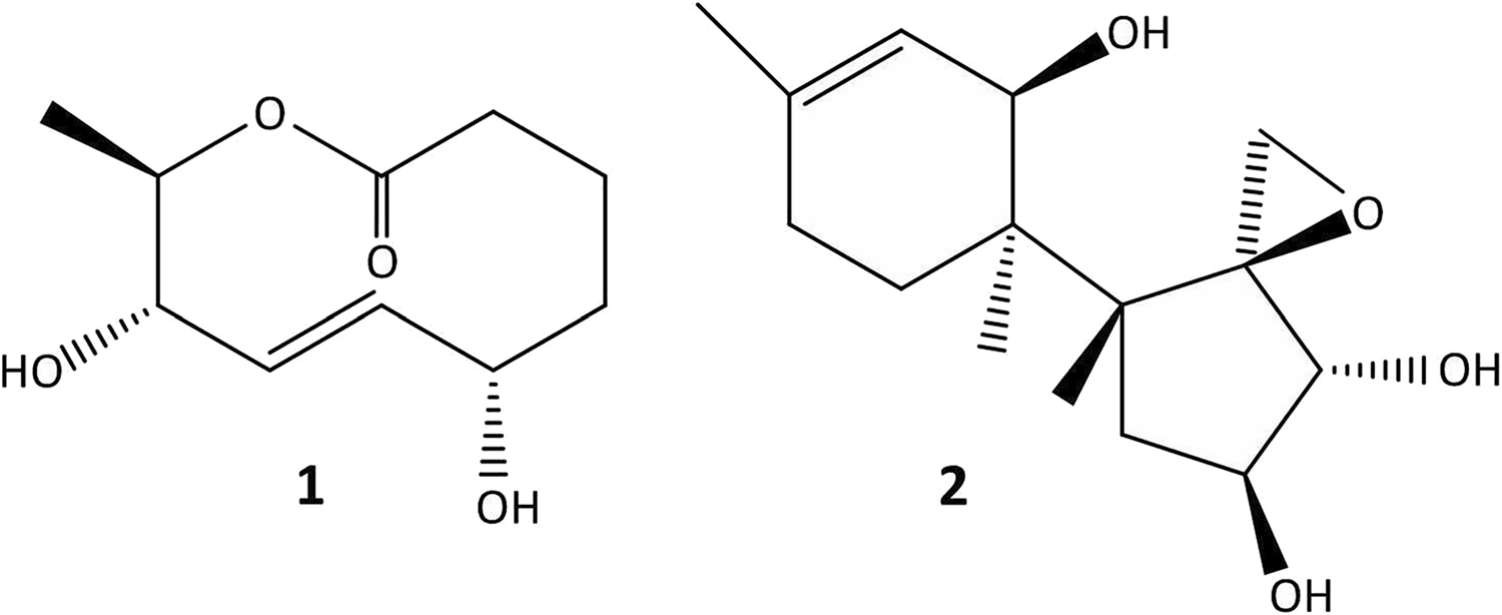
Chemical structure of compounds **1** (8-hydroxy aspinolide A) and **2** (3,11-diepiisotrichotriol) isolated from culture broths of Δasp1.9 mutant

In addition to data presented above, the analysis of the large-scale chromatographic fractions collected from extracts of Δ*asp1* mutant cultures indicated production of a major metabolite (compound **1**) in one of the fractions. Total purification by HPLC (t_r=_ 37 min, Hex:Ea 50%, Flux of 1 mL/min, analytical silica gel column) and subsequent spectroscopic analysis characterized **1** as a novel aspinolide analog called 8-hydroxy aspinolide A (Fig. 2). Furthermore, **1** was detected in culture extracts of the Δ*asp1* mutant but was absent in culture extracts of the Δ*asp2* mutant and wild-type strain (Table 2).

To determine the structure, purified **1** was subjected to NMR spectroscopy as described in the “Materials and methods” section. The resulting ^1^H NMR and ^13^C NMR spectroscopic data show a pattern of signals characteristic of aspinolides but with significant differences (Table 3). Specifically, the ^1^H NMR spectrum resembled that of aspinolide D (Figure S1) but lacked signals corresponding to C-4 ester at C-8, indicating its underlying carbon skeleton similar in structure to the aspinolide lactone ring. The associated H-8 resonance of compound **1** now appeared at δ 3.88 ppm and the characteristic signals of the proton geminal to the hydroxyl group at C-5 (δ 68.3 ppm) carbon appeared at δ 4.49 (d(br), *J* = 6.0 Hz). The ^13^C- NMR spectrum displayed signals corresponding to the double bond at C-6 (δ 130.1 ppm) and C-7 (δ 135.8 ppm). We also detected heteronuclear multiple bond correlation (HMBC) signals between H-9 and C-1, C-8; H-2 and C-1, C-3, a﻿n﻿d C-4; and between H-5 and C-3, C-4, C-6, and C-7. The position of the double bond was confirmed by signals between H-6 and C-5, C-7 and between H-7 and C-5, C-8, and C-9. This relationship to aspinolide D was supported by COSY and HSQC experiments confirming the proposed structure for compound **1** as 8-hydroxy aspinolide A (Fig. 2).

**Table 3:** Spectroscopic data for 8-hydroxy aspinolide A (**1**) (^1^H NMR (CDCl_3_, 500 MHz), ^13^C NMR (CDCl_3_, 125 MHz)

Position	Proton	δ ^1^H (Hz)	δ^13^C	HMBC
**1**			176.7	
**2**	H-2a	2.45 (dd, *J* = 15.0, 8.7 Hz)	36.1	C1, C3, C4
	H-2b	1.96 (m)		C1, C3, C4
**3**	H-3a	2.07 (m)	18.4	C1, C2, C4, C5
	H-3b	1.7 (ddd, *J* = 15.0, 7.0, 4.4 Hz)		C1, C2, C5
**4**	H-4a	2.05 (m)	36.7	C2, C3
	H-4b	1.52 (m)		C2, C3
**5**	H-5β	4.49 (d(br), *J* = 6.0 Hz)	68.3	C3, C4, C6, C7
**6**	H-6β	5.52–5.59 (m)	130.1	C5, C7
**7**	H-7α	5.58–5.64 (m)	135.8	C5, C8, C9
**8**	H-8β	3.88 (dd, *J* = 8.7, 7.0 Hz)	79.7	C6, C7, C9, C10
**9**	H-9α	4.95 (dq, *J* = 8.7, 6.4 Hz)	75.2	C1, C8
**10**	H-10	1.42 (d, *J* = 6.4 Hz, 3H)	16.9	C8, C9

The accumulation of **1** in cultures of the Δ*asp1* mutant combined with its absence in cultures of the Δ*asp2* mutant and wild-type strain indicated that the Δ*asp1* mutant can produce the 10-member lactone ring that forms the aspinolide backbone but not the 8-butenoyl substituent (Fig. 1A). This is consistent with the *asp1*-encoded PKS (ASP1) catalyzing synthesis of the diketide precursor of the 8-butenoyl substituent. Furthermore, the absence of both **1** and previously described aspinolides in culture extracts of the Δ*asp2* mutant is consistent with the *asp2*-encoded PKS (ASP2) catalyzing synthesis of the polyketide precursor of the aspinolide backbone.

#### Production of harzianum A

**Fig. 3:**
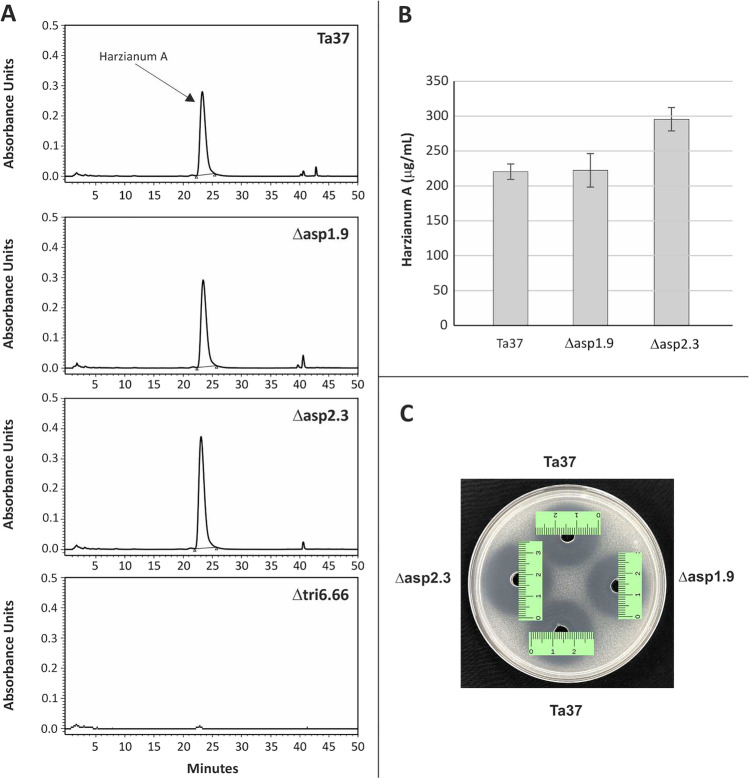
**A** HPLC chromatograms showing the HA peak detected in 48 h PDB cultures of Ta37 (wild type), Δasp1.9, Δasp2.3, and Δtri6.66*. **B** Quantification of HA in Ta37 and Δ*asp1* and Δ*asp2* mutants (n = 2). **C** Antibiogram of ethyl acetate extracts of 48 h PDB cultures of wild-type *T. arundinaceum* (Ta37) and the mutant strains Δasp1.9 and Δasp2.3 against *Kluyveromyces marxianus* CECT 1018. *Δtri6.66 is a mutant deleted on tri6** gene that produces trace amounts of HA (Lindo et al., 2018) and was used in this study for comparative purposes. **tri6 gene encodes a Cys2His2-Zn finger transcription factor that regulates expression of *tri* genes and is thereby essential for trichothecene biosynthesis

To examine whether the change in aspinolide production in the Δ*asp1* and Δ*asp2* mutants affected HA production, we quantified the amount of HA present in 48 h PDB cultures of the mutants and wild-type strain. The Δ*asp2* mutant produced significantly more HA (296.3 ± 17.0 μg/mL) than the wild type (220.1 ± 11.1 μg/mL), which represents an increase of 34% (Fig. 3A, B). The amount of HA produced by the Δ*asp1* mutant (222.3 ± 24.0 μg/mL) was similar to what was produced by the wild-type strain (Fig. 3A, B).

#### Production of a novel isotrichotriol epimer

Chromatographic fractions from culture extracts of the Δ*asp1* and Δ*asp2* mutants contained a second unknown metabolite (compound **2**). Purification by HPLC and subsequent spectroscopic analysis indicated that **2** is a C-3, C-11 diepimer of isotrichotriol. **2** was not detected in culture extracts of the wild-type strain (Table 2, Fig. 2).

To determine the structure, purified **2** was subjected to high-resolution mass spectrometry (HRMS) and NMR. These data indicated that **2** has a molecular formula of C_15_H_24_O_4_. The ^1^H NMR signals resembled those of a sesquiterpene (Table S4) and included signals characteristic of an oxiranic methylene at δ 2.99 (H-13a) and 2.80 (H-13b) (d, *J* = 4.1 Hz) and three geminal protons to hydroxyl groups, indicating that **2** was a trihydroxylated sesquiterpene with an epoxide. Although the ^1^H and ^13^C NMR signals of **2** were similar to those of tricho-9-ene-2α,3α,11α-triol (isotrichotriol) (McCormick et al. 1989) and included the characteristic signals of a double bond at C-9:C-10 and the three protons germinal to hydroxyl groups at C-2, C-3, and C-11, but there were some significant differences. The signal assigned to H-11 was shielded at δ 3.58 while the signal of H-3 was deshielded at δ 4.40, with the H-2 signal appearing at δ 3.66 (Table S4). The stereochemistry was assigned based on nuclear Overhauser effect (NOE) experiments. Irradiation of H-2 enhanced signals corresponding to H-13a and H-4β, while α configuration for H-11 and H-3 was established by irradiation of H-15, which produced NOE effects at H7α, H-8α, H-11, and H-3. On the other hand, irradiation of H-3 enhanced signals corresponding to H-11, H-15, and H-4α. Consequently, the compound was assigned the structure **2**. All the carbon and their associated proton signals were assigned using the COSY, HSQC, and HMBC spectra leading to the structure and stereochemistry of compound **2**, which was named 3,11-diepiisotrichotriol (**2**). HRMS(ESI^+^) for compound **2**: calcd. for C_15_H_24_O_4_Na [M + Na]^+^ 291.1572, found 291.1571. **2** (or 3,11-diepiisotrichotriol) is an epimer of isotrichotriol, an intermediate in biosynthesis of 3-oxygenated trichothecenes produced by *Fusarium* spp., but not an intermediate in biosynthesis of HA (Gutiérrez et al. 2020; Proctor et al. 2020).

#### Production of primary metabolites

In previous works, changes in the levels of squalene and ergosterol were observed in *T. arundinaceum* strains blocked in trichothecene biosynthesis (Malmierca et al. 2013). In the current study, we quantified the levels of squalene and ergosterol produced in cultures of the Δ*asp1* and Δ*asp2* mutants and the wild-type strain to determine if blocking aspinolide biosynthesis affected their levels. We found no significant changes in levels of either squalene or ergosterol in the Δ*asp1* and Δ*asp2* mutants as compared to the wild type (Table S5). In addition, the analysis of the low polarity chromatographic fractions collected from the extracts of the culture filtrate of these mutants indicated that they contained high levels of fatty acids (Table 2). No difference in growth was observed in the two mutants compared with the wild-type strain.

### Effect of Δ*asp1* and Δ*asp2* gene deletion on the antifungal activity

The contribution of aspinolide production to the antifungal activity of *T. arundinaceum* was assessed using the yeast *K. marxianus* and the filamentous fungus *R. solani*. The antifungal activity of pure AspB and AspC was first evaluated against these two fungi. AspC inhibited both fungi, while AspB failed to inhibit either fungus (Figure S8).

In subsequent assays with *K. marxianus*, ethyl acetate extracts of 48 h PDB cultures of Δ*asp1* and Δ*asp2* mutants and the wild-type progenitor strain were examined. Extracts of the Δ*asp2* mutant inhibited growth of *K. marxianus* more than extracts of the Δ*asp1* mutant and the wild type. There were no differences in the inhibition caused by culture extracts of the Δ*asp1* mutant and wild type (Fig. 3C). The greater inhibition observed by extracts of the Δ*asp2* mutant is consistent with the higher levels of HA in the extracts compared to extracts of Δ*asp1* mutant or the wild type (Fig. 3A, B).

In assays with *R. solani*, plates were incubated for 7 and 10 days after pathogen placement in the center of the plate, after removal of the cellophane membranes where the *T. arundinaceum* strains were previously grown for 24 h. Mean percent radial inhibition caused by the Δ*asp2* mutant was significantly higher than that caused by the Δ*asp1* mutant and wild type at both 7 and 10 days. By contrast, inhibition caused by Δ*asp1* mutant and wild type was not significantly different at either day (Table S6). These results indicate that the Δ*asp2* mutant inhibits the growth of *R. solani* more than the Δ*asp1* mutant and the wild-type strain (Figure S9).

### Transcription analysis of the genomic region with *asp1* and *asp2*

**Fig. 4:**
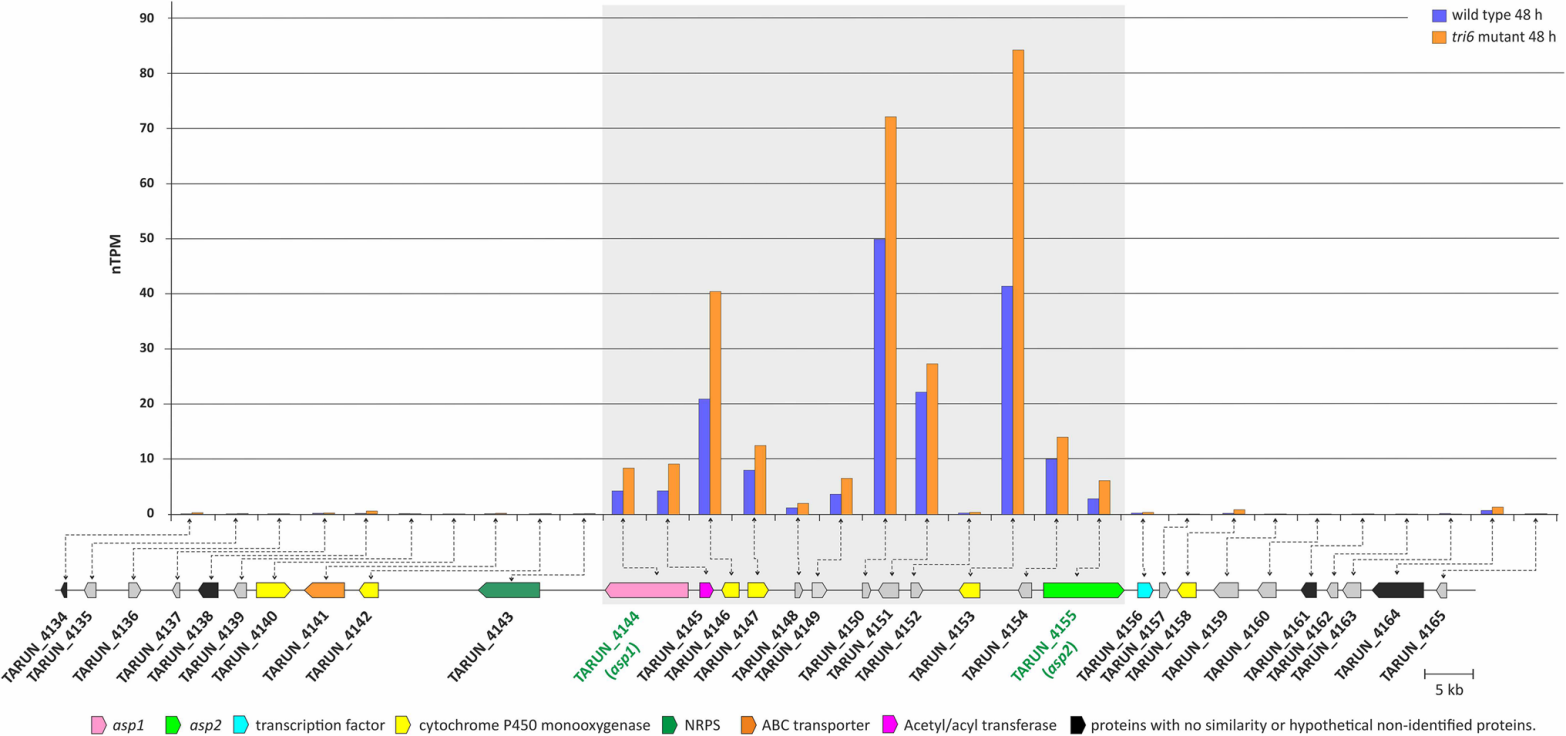
Upper panel. Expression of genes flanking *asp1* (TARUN_4144) and *asp2* (TARUN_4155) in a wild-type (blue bars) and a *tri6* mutant (orange bars) strain of *T. arundinaceum* grown for 48 h in PDB medium. nTPMs, transcripts per million (TPM) of total reads of each gene normalized against TPM of the actin gene (housekeeping) of each sample. Lower panel. Graphic representation of the genomic region showing positions of genes analyzed in the upper panel. Genes and their direction of transcription are indicated by arrows. The positions of *asp1* and *asp2* are highlighted using green text. Predicted function for each gene is indicated by arrow color and their associated codes (Table 1, Table S3). Predicted functions for all genes included in this genomic region are indicated in Table S7. Numeric data used to generate this histogram are included in Table S8

In the current analysis, we used RNAseq to assess expression of the 32 genes (locus tags TARUN_4134 to TARUN_4165) in the genomic region in which *asp1-asp2* are located in the wild-type *T. arundinaceum* and a *tri6*-deletion mutant strain Δtri6.66). The 32 genes include *asp1* and *asp2*, 10 genes located between them, 10 genes downstream of *asp1*, and 10 genes downstream of *asp2* (Table S7). The rationale for including the *tri6* mutant in this analysis was that in a previous study (Lindo et al., 2018) it produced higher levels of aspinolides than its wild-type progenitor strain (Ta37) and, therefore, transcription of aspinolide biosynthetic genes could be higher in the mutant than in the wild type. The RNAseq analysis in the current study revealed that *asp1*, *asp2*, and the 10 genes in between them exhibited similar patterns of expression in that they were more highly expressed in the *tri6* mutant than in the wild type, even though there were marked differences in the levels of expression among some genes (Fig. 4, Table S8). In contrast, the 10 genes downstream of *asp1* and the 10 genes downstream of *asp2* exhibited low levels of expression, and their expression levels in the *tri6* mutant were not consistently higher than in the wild type. The greater expression of *asp1* and *asp2* in the *tri6* mutant relative to the wild type was consistent with the previously reported higher levels of aspinolides produced by the mutant (Lindo et al., 2018). Together, these data provide evidence that *asp1*, *asp2*, and the 10 genes located between them (i.e., TARUN_4144 and TARUN_4155) are coregulated and, therefore, could be an aspinolide biosynthetic gene cluster.

### Identification of aspinolide gene clusters orthologs and production of aspinolides in other *Trichoderma* species

BLASTp analysis was performed using the *T. arundinaceum* ASP1 (TARUN_4144) and ASP2 (TARUN_4155) homologs as queries against an in-house database containing protein sequences predicted to be encoded by genes in the genome of 35 evolutionary diverse *Trichoderma* species (Gutiérrez et al. 2021; Kubicek et al. 2019).

**Fig. 5:**
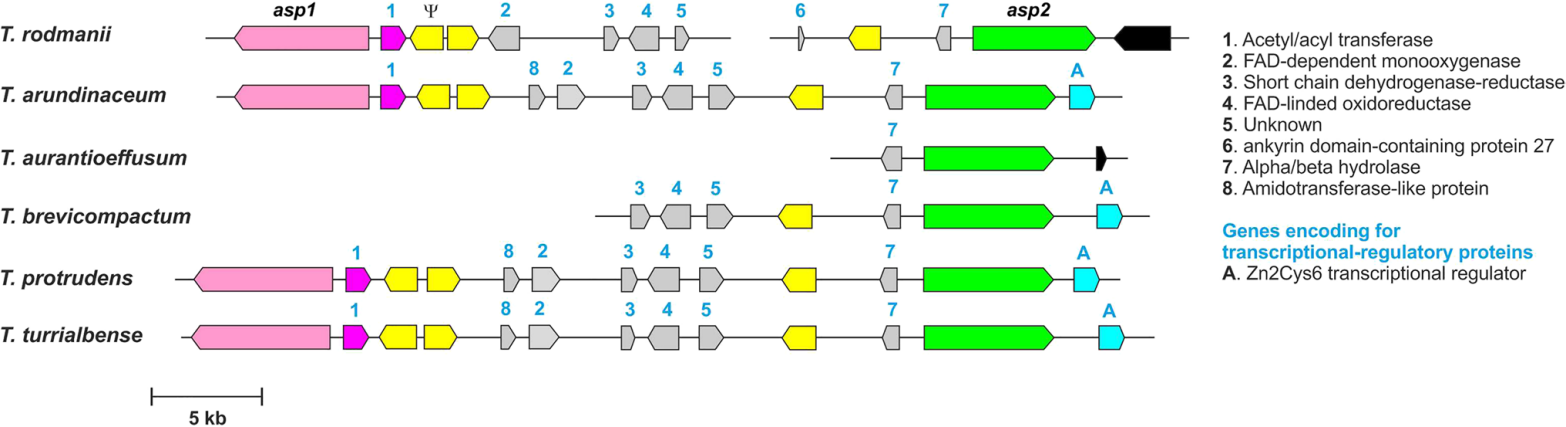
Occurrence of intact and partial homologs of the proposed aspinolide biosynthetic gene cluster in *Trichoderma* species in the Brevicompactum clade. Predicted functions of proteins encoded by these genes are indicated at the right. Ψ indicates a pseudogene. Color codes used to illustrate predicted function of each gene were as described in the legend for Fig. 4

Putative *asp1* homologs were identified in three species belonging to the Brevicompactum clade: *T. rodmanii*, *T. protrudens*, and *T. turrialbense*. Putative *asp2* homologs were identified in these three species as well as two other species in the Brevicompactum clade: *T. aurantioeffusum* and *T. brevicompactum*. Analysis of the genomic region flanking the *asp1* and *asp2* gene homologs in two species, *T. protrudens* and *T. turrialbense*, indicated that they shared a high degree of sequence identity and synteny to the SM cluster predicted by antiSMASH that included *asp1* and *asp2* in *T. arundinaceum* (Fig. 5). In contrast, the putative *asp1* and *asp2* homologs in *T. rodmanii* were located on two different contigs. Analysis of the regions flanking *asp1* and *asp2* revealed putative homologs of most genes flanking *asp1* and *asp2* in *T. arundinaceum* except that homologs to TARUN_4148 and TARUN_4156 were missing and the homolog of TARUN_4146 is likely nonfunctional (Fig. 5). Analysis of genomic region flanking *asp2* in *T. aurantioeffusum* and *T. brevicompactum* indicated that they share a high degree of similarity spanning one and five predicted genes, respectively, with genes flanking *asp2* in *T. arundinaceum*. Finally, no genes belonging to this cluster were found in *T. margaretense*, another species belonging to the Brevicompactum clade (Fig. 5) Thus, based on sequence similarity, shared synteny between these *Trichoderma* species, and co-expression in *T. arundinaceum*, we propose that the *asp1* and *asp2* are part of a gene cluster that includes 12 genes.

#### Production of aspinolides by *Trichoderma* species in the Brevicompactum clade

**Fig. 6:**
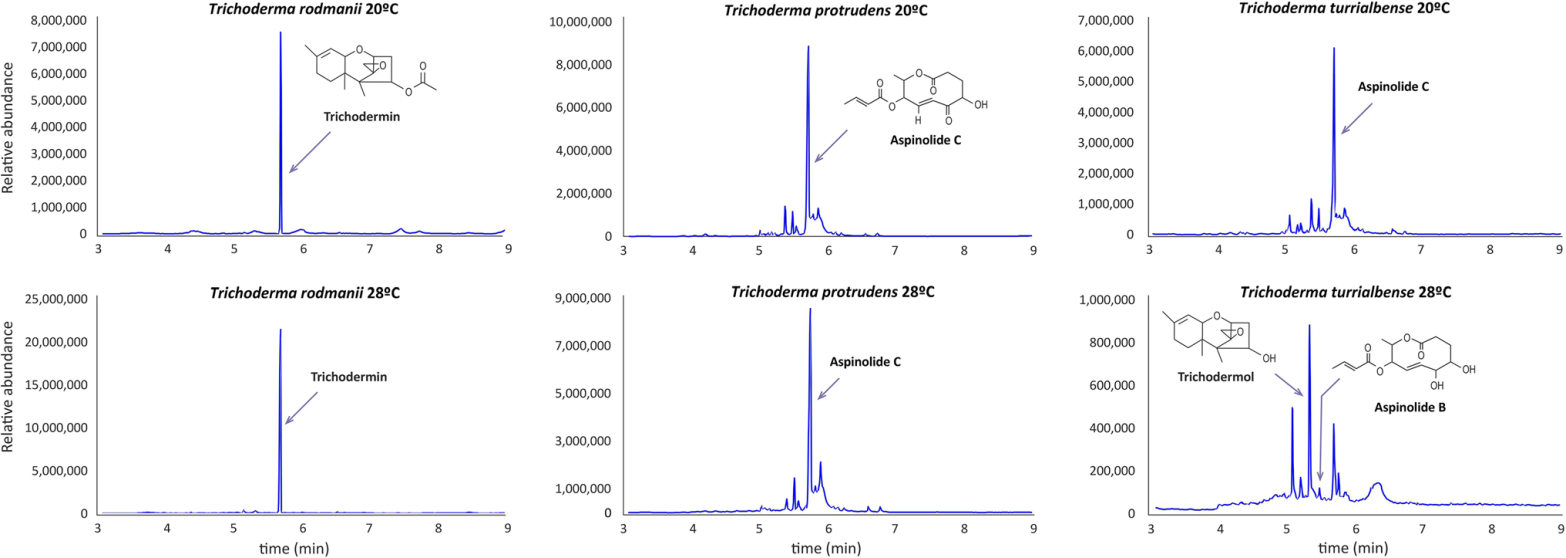
GC-MS analysis of extracts and identification of aspinolides and trichothecene analogs produced by *T. rodmanii*, *T. protrudens*, and *T. turrialbense*, in cultures grown at 20 and 28 °C. Note the scale at the Y-axis is different on each graphic

To determine whether the presence of *ASP* clusters in *T. rodmanii, T. protrudens*, and *T. turrialbense* correlates with aspinolide production, all three strains were grown for 7 days, at either 20 °C or 28 °C. GC-MS analysis of culture extracts indicated that both *T. protrudens* and *T. turrialbense* can produced aspinolides (B or C) at both assayed temperatures with the highest production observed at 20 °C. In addition, the sesquiterpene intermediate metabolite trichodermol was also detected in the *T. turrialbense* 28 °C extracts (Fig. 6). The detection of the aspinolides was fully consistent with the presence of an intact aspinolide gene cluster in both strains. On the other hand, no aspinolides were detected in *T. rodmanii* extracts. Furthermore, a significant amount of the trichothecene analog trichodermin was detected in *T. rodmanii* extracts at both temperatures (Fig. 6). The failure of *T. rodmanii* to produce aspinolide suggests that the genetic differences observed between the aspinolide clusters in *T. rodmanii* versus *T. arundinaceum*, *T. protrudens*, and *T. turrialbense* are critical for aspinolide production. These differences can be summarized as follows: (i) pseudogenization of a cytochrome P450-monooxygenase gene (labeled as Ψ in Fig. 5) and (ii) absence of an amidotransferase-like gene (labeled as 8 in Fig. 5). In addition, it is noteworthy that *T. rodmanii* is missing a gene encoding a Zn2Cys6 transcriptional regulator that is adjacent to *asp2* (labeled A in Fig. 5). The close proximity of gene A to *asp2* suggests a potential role in aspinolide synthesis.

### Phylogenetic relationships and distribution of *asp1* and *asp2*

**Fig. 7:**
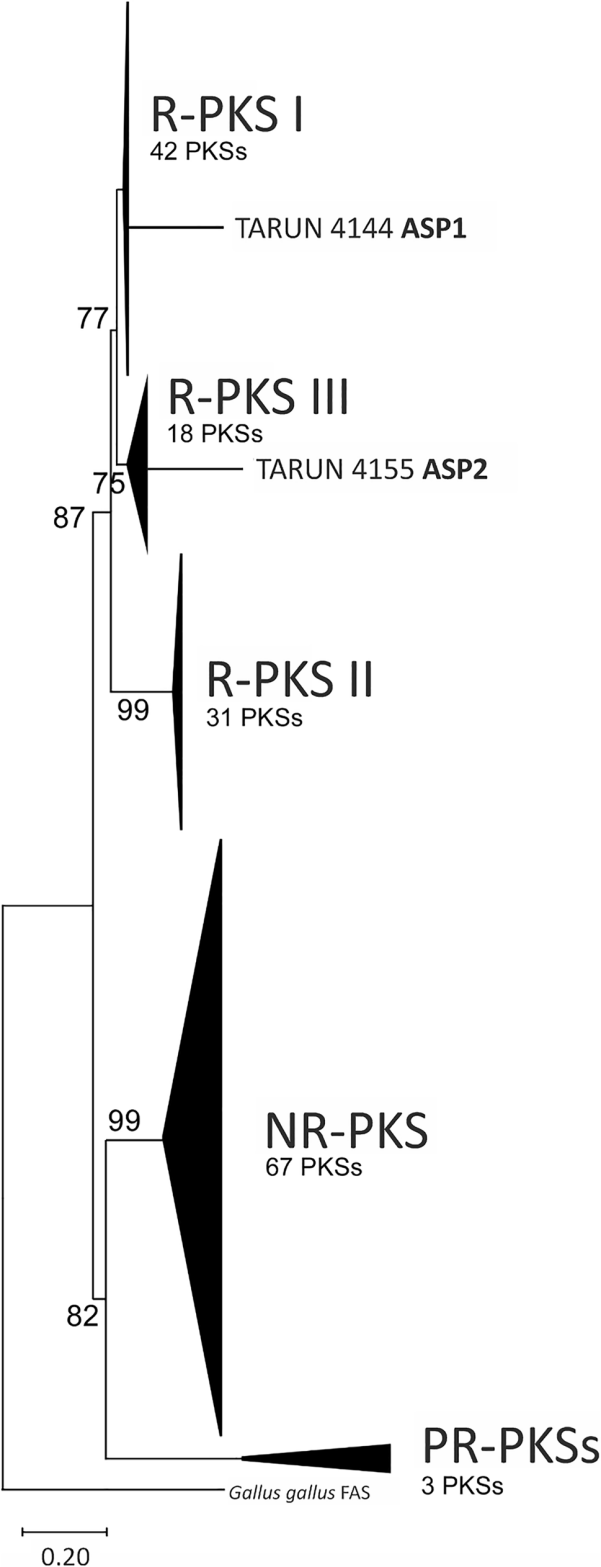
Phylogenetic analysis resolved ASP1, ASP2, and 159 predicted fungal PKS proteins with known functions (Brown et al. 2022) into three previously described groups represented by five clades. The three groups are non-reducing PKSs (NR-PKSs), reducing PKSs (R-PKSs), and partial reducing (PR-PKSs). The R-PKS group has been further divided into three subgroups based on domain functionality and phylogenetic relationships: R-PKS I, R-PKS II, and R-PKS III. The fatty acid synthase from *Gallus gallus* served as an outgroup

Phylogenetic analysis of predicted ASP1, ASP2, and 159 fungal PKSs with known functions were resolved into three previously described clades of fungal PKSs: non-reducing PKSs (NR-PKSs), reducing PKSs (R-PKSs), and partial reducing PKSs (PR-PKSs) (Fig. 7). The R-PKSs were further resolved into three smaller clades: R-PKS I, R-PKS II, and R-PKS III. ASP1 was nested within the R-PKS I clade, and ASP2 was nested within the R-PKS III clade (Fig. 7).

BLASTx analysis using the *T. arundinaceum asp1* and *asp2* homologs as queries against the GenBank/NCBI nonredundant fungal database identified homologs of both genes in other fungi (Figure S10). We focused our analysis on fungi corresponding to the 10 best hits in the BLASTx analysis in which *T. arundinaceum asp1* was the query (*E*-value 0.0; 50–61% amino acid sequence identity over 97–98% of the query sequence) because each of these hits were for fungi that also had *asp2* homologs with 48–61% amino acid sequence identity to *T. arundinaceum* ASP2. In contrast, when *T. arundinaceum asp2* was used as a query sequence in BLASTx, the top 10 hits were for fungi that only had relatively distantly related homologs of *T. arundinaceum* ASP1 (< 36% amino acid sequence identity). The fungi corresponding to the 10 best hits in the *asp1* BLASTx analysis were from two orders within class Sordariomycetes: Sordariales and Xylariales. The one exception to this was *Aspergillus melleus*, which like *A. ochraceus* is in class Eurotiomycetes and order Eurotiales. In five of the fungi, the *asp1* and *asp2* homologs were located near one another on the same contig and, therefore, could be in the same biosynthetic gene cluster. In the other five fungi, the two genes were located on different contigs. However, in some of the latter fungi (e.g., *Madurella mycetomatis*), the genes were located on small contigs (< 50 kb) and/or at the ends of contigs. In these fungi, it is possible that the *asp1* and *asp2* homologs are located near one another on a chromosome.

In trees inferred by maximum likelihood analysis of the predicted amino acid sequences of ASP1 and ASP2 homologs, *Trichoderma* sequences formed a well-supported and exclusive clade (Figure S10). *Aspergillus ochraceus* and *A. melleus* sequences also formed a well-supported and exclusive clade in both trees. In the ASP2 tree, the *Aspergillus* sequences were nested within a well-supported clade that excluded the *Trichoderma* sequences (Figure S10). In the ASP1 tree, however, the relationships of the *Aspergillus* sequences to the *Trichoderma* sequences were not resolved except that sequences from each of these two genera were resolved as exclusive clades (Figure S10). Nevertheless, in both trees, the *Aspergillus* sequences were nested within a well-supported clade (bootstrap values = 100) that otherwise consisted of sequences from fungi in class Sordariomycetes.

## Discussion

### Evidence for role of *asp1* and *asp2* in aspinolide biosynthesis

Aspinolide production in *T. arundinaceum* was first discovered in studies in which the antifungal activity of the fungus was retained following loss of production of the antifungal metabolite HA (Lindo et al. 2019a, 2019b; Malmierca et al. 2015). These studies also provided evidence that HA and AspC may have redundant roles in the ecology of the fungus in that they both have antifungal activity and can induce expression of plant defense-related genes. We initiated the current study to identify aspinolide biosynthetic genes as a means to further investigate the role of aspinolide production in the ability of *T. arundinaceum* to inhibit growth of other fungi. We obtained multiple lines of evidence that the PKS genes *asp1* and *asp2* are part of an aspinolide biosynthetic gene cluster. First, evidence from previous studies indicates that aspinolide synthesis requires two PKS genes, and *asp1* and *asp2* are the only two *T. arundinaceum* PKS genes that have closely related homologs in *A. ochraceus*, a distantly related, aspinolide-producing fungus. Second, deletion of *asp1* and *asp2* in *T. arundinaceum* blocked wild-type aspinolide production in *T. arundinaceum*. In-depth biochemical analyses indicated that the *asp2* mutant does not produce any aspinolides while the *asp1* mutant produced a novel aspinolide analog that lacks the 8-butenoyl substituent. Third, in both *T. arundinaceum* and *A. ochraceus*, *asp1* and *asp2* are located within an antiSMASH-predicted gene cluster. The gene content of the cluster in the two species differs, but in addition to *asp1* and *asp2*, some genes that are common to the two homolog clusters are predicted to encode enzymes with the types of activities predicted to be necessary for aspinolide biosynthesis (see below for proposed biosynthetic pathway). Finally, two other *Trichoderma* species that have homologs of the *T. arundinaceum* 12-gene cluster produced aspinolides, while a third species with only a partial cluster did not (Fig. 5, Fig. 6).

The finding that ASP1 and ASP2 are required for aspinolide biosynthesis adds to the growing list of fungal SMs whose synthesis requires two PKSs. For example, the synthesis of zearalenone by *Fusarium* species (Gaffoor and Trail 2006), botcinic acid by *Botrytis cinerea* (Dalmais et al. 2011), and lovastatin by *Aspergillus terreus* (Kennedy et al. 1999) all require two PKSs. Furthermore, a recent genus-wide analysis of PKSs indicated that approximately 8% of polyketide-derived SMs produced by *Fusarium* likely require two PKSs for their formation (Brown et al. 2022).

### Changes in aspinolide and HA production

The increase in the levels of HA produced by the Δ*asp2* mutant in the current study (Fig. 3) is reminiscent of increases in aspinolide production in previous studies on *T. arundinaceum* strains in which HA production was blocked by deletion of *tri* genes. In one study, AspB and AspC were not detected in cultures of wild-type *T. arundinaceum* but were present at high levels in cultures of the Δ*tri5* mutant, which does not produce HA or HA biosynthetic intermediates (Malmierca et al. 2015). In another study, levels of AspC were over 10 times higher in cultures of Δ*tri18* mutants, which are blocked in HA production but produce the trichothecene biosynthetic intermediate trichodermin, than in cultures of wild-type *T. arundinaceum* (Lindo et al. 2019a). The 34% increase in HA levels produced by the Δ*asp2* mutant constitutes a relatively small increase compared to the marked increases (> 10 times) in aspinolide production observed in at least some HA-nonproducing Δ*tri* mutants (Lindo et al. 2019a; Malmierca et al. 2015).

In contrast to the Δ*asp2* mutant, the Δ*asp1* mutant did not exhibit a detectable increase in HA production compared to wild-type *T. arundinaceum* (Fig. 3). Because the Δ*asp1* mutant produced 8-hydroxy aspinolide A, a presumed aspinolide biosynthetic intermediate, the latter result raises the possibility that the increased HA production requires a complete loss of production of all aspinolide analogs. This contrasts the situation with Δ*tri* mutants, where deletion of *tri18*, which results in the formation of trichothecene analog trichodermin, caused marked increases in AspC production (Lindo et al. 2019a). The genetic and physiological mechanisms by which a loss of HA production leads to increased aspinolide production and loss of aspinolide production leads to increased HA production remain to be determined.

### Antifungal activity of Δ*asp1* and Δ*asp2* mutants

Both the Δ*asp1* and Δ*asp2* mutants retained antifungal activity against *K. marxianus* and *R. solani* (Fig. 3C, Figure S9). In fact, the Δ*asp2* mutant had slightly higher than wild-type levels of antifungal activity against both *K. marxianus* and *R. solani*. This increased antifungal activity coincided with a 34% increase in levels of HA produced by the Δ*asp2* mutant. Retention of antifungal activity of *T. arundinaceum* mutants blocked in production of HA was attributed to increased production of aspinolides. Thus, it is possible that retention of antifungal activity of the Δ*asp2* mutant resulted from an increase in the production of HA by the mutant. These data add to existing information indicating that HA and aspinolides have redundant bioactive roles in a manner analogous to the redundant roles of botcinic acid and botrydial analogs in the biology of *B. cinerea* (Dalmais et al. 2011). However, we cannot rule out the possibility that another metabolite(s) contributes to the antifungal activity of *T. arundinaceum* mutants that are blocked in aspinolide or HA production. In order to address this possibility, we are currently attempting to generate double Δ*asp2*-Δ*tri5* mutants, which should not produce aspinolides, HA, or any other trichothecene analogs.

Our finding that the level of antifungal activity of the Δ*asp1* mutant was similar to the wild-type was unexpected because the mutant does not produce aspinolide C and it does not exhibit increased levels of HA production that might compensate for the absence of aspinolide C. Without further experimentation, we can only speculate why the Δ*asp1* mutant exhibited wild-type antifungal activity. One possibility is that the 8-hydroxy aspinolide A produced by the Δ*asp1* mutant has antifungal activity that compensates for the absence of aspinolide C. This possibility could be addressed by determining whether 8-hydroxy aspinolide A is antifungal. A second possibility is that the antifungal activities of aspinolide C and HA are not additive or synergistic when produced at wild-type levels. An improved understanding of why loss of aspinolide production increases HA production, and vice versa, could help address this possibility. A third possibility is that *T. arundinaceum* produces another antifungal metabolite(s) that compensates for the absence of aspinolide C production in the Δ*asp1* mutant. As noted above, we are currently addressing this possibility by generating double *asp2*-*tri5* mutant that should not produce aspinolides or trichothecenes.

### Formation of 3,11-diepiisotrichotriol

To our knowledge, production of 3,11-diepiisotrichotriol by the Δ*asp1* and Δ*asp2* mutants in the current study is the first report of this metabolite. Production of 3,11-diepiisotrichotriol by *T. arundinaceum* is intriguing because, although isotrichotriol is an intermediate in the biosynthesis of the 3-oxygenated trichothecenes produced by *Fusarium* species, it is not an intermediate in biosynthesis of trichothecene analogs produced by *T. arundinaceum* (Proctor et al. 2020). Furthermore, the epimer of the *Fusarium* isotrichotriol has a different stereochemistry than the isotrichotriol epimer produced by the Δ*asp1* and Δ*asp2* mutants of *T. arundinaceum*. This report of 3,11-diepiisotrichotriol by the Δ*asp1* and Δ*asp2* mutants will facilitate the determination of whether other strains of *T. arundinaceum* and other *Trichoderma* species can produce the metabolite.

Isotrichodiol is the trichothecene biosynthetic intermediate formed by *T. arundinaceum* that is most similar in structure to isotrichotriol. These two metabolites differ in structure only by the presence (isotrichotriol) and absence (isotrichodiol) of a hydroxyl at C-3. Previous studies indicate that this structural difference at C-3 results from differences in activities of TRI4 homologs in *Trichoderma* and *Fusarium* species. *Trichoderma* TRI4 catalyzes oxygenation of the terpene trichodiene at three positions to form isotrichodiol (Cardoza et al. 2011), while the *Fusarium* TRI4 catalyzes oxygenation of trichodiene at four positions to form isotrichotriol (McCormick et al. 2006; Tokai et al. 2007). It is not clear whether the formation of the 3-hydroxyl of 3,11-diepiisotrichotriol is catalyzed by a heretofore unrecognized activity of the *T. arundinaceum* TRI4 homolog or some other enzyme. The low levels of 3,11-diepiisotrichotriol produced by the Δ*asp1* and Δ*asp2* mutants of *T. arundinaceum* suggests that whichever enzyme is responsible it has low levels of trichodiene 3-hydroxylase activity.

### Distribution and phylogenetic relationships of aspinolide biosynthetic genes

The 35 *Trichoderma* genome sequences included in the genome survey in the current study represented a wide breadth of the phylogenetic diversity that exists within the genus *Trichoderma*. Thus, the detection of putative aspinolide biosynthetic gene cluster only in members of the Brevicompactum clade suggests that the cluster has a limited distribution within the genus. The occurrence of cluster homologs with all 12 putative aspinolide biosynthetic genes in some species of the Brevicompactum clade and homologs with only some of the genes in other species of the clade further suggests that an ancestral species of the Brevicompactum clade had an intact aspinolide gene cluster and that as the ancestor diverged into multiple species, an intact cluster was retained in *T. arundinaceum*, *T. protrudens*, and *T. turrialbense* but partially or completely degenerated in other species.

The presence of closely related homologs of genes in distantly related fungal species combined with absence of the genes in taxa closely related to either species can be a sign of horizontal gene transfer (Campbell et al. 2012; Slot and Rokas 2011). Thus, horizontal gene transfer provides a possible explanation for the presence of the aspinolide cluster in *A. ochraceus* and the Brevicompactum clade of *Trichoderm*a. However, phylogenetic analysis of ASP1 and ASP2 homologs from other fungi suggest that the putative horizontal transfer might not have involved both *Aspergillus* and *Trichoderma* species (Figure S10). In the ASP1 and ASP2 trees, the *Aspergillus* sequences were nested within a well-supported clade that otherwise consisted only of sequences from the Sordariomycetes. This suggests that the direction of transfer was from a species of the Sordariomycetes to *Aspergillus*, presumably a common ancestor of *A. melleus* and *A. ochraceus*. The poor resolution of the relationship of the *Aspergillus* clade and other clades in the ASP1 tree precluded hypotheses about which fungus served as a donor in the putative transfer to *Aspergillus*. In the ASP2 tree, by contrast, there was high bootstrap support to indicate that the *Aspergillus* sequences are most closely related to a *Monosporascus* sp. (order Xylariales) sequence and a clade consisting of *Chaetomium* sp. and *Madurella mycetomatis* (order Sordariales) sequences. This suggests two potential donors: *Monosporascus* sp. or a common ancestor of *Chaetomium* sp. and *M. mycetomatis*. Identification of aspinolide biosynthetic gene cluster homologs in additional fungi and phylogenetic analyses that more rigorously assess potential horizontal transfer events (Slot and Rokas 2011; Villani et al. 2019) should provide further insight into the putative horizontal transfer of the aspinolide cluster.

### Aspinolide biosynthetic pathway

**Fig. 8:**
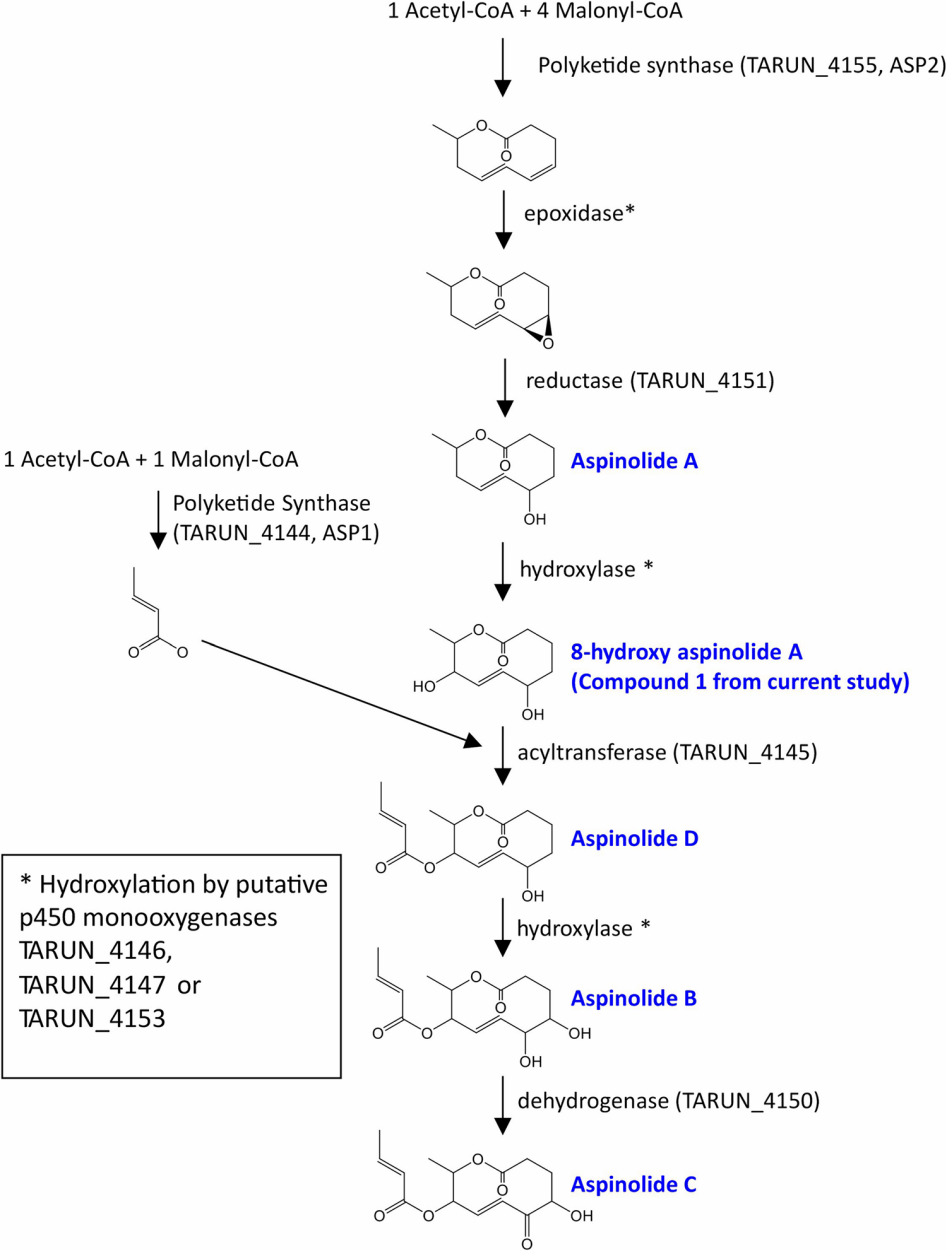
Proposed aspinolide biosynthetic pathway. The scheme proposed by Fuchser and Zeeck (1997) was updated using DNA sequence, gene function, and biochemical data reported in the current study. The proposed catalytic activity for the structural changes depicted are indicated at the right of each arrow. Names of aspinolide analogs are indicated in blue type

We have proposed an updated aspinolide biosynthetic pathway (Fig. 8) based on three lines of evidence: (a) the pathway previously proposed by Fuchser and Zeeck (1997); (b) the results of biochemical analyses of the Δ*asp1* and Δ*asp2* mutants (current study); and (c) the predicted functions, based on sequence homology, of genes in the putative aspinolide biosynthetic gene clusters in *T. arundinaceum* and *A. ochraceus* (current study). The proposed pathway begins with condensation of one acetate and four malonate units followed by cyclization to form the 10-member lactone ring that is the common backbone of all aspinolide analogs. The inability of the Δ*asp2* mutant to produce aspinolides indicates that the ASP2 PKS catalyzes these reactions. The 10-member lactone ring then undergoes a series of reactions that yield multiple aspinolide analogs. The 5-hydroxy intermediate, aspinolide A, has not been detected in *Trichoderma* cultures but it has been detected in *A. ochraceus* cultures (Fuchser and Zeeck 1997). The predicted functions of the proteins encoded by some genes in the putative *T. arundinaceum* aspinolide gene cluster are consistent with the proposed pathway reactions. For example, the pathway includes three oxygenation reactions (4,5-epoxidation, 8-hydroxylation, and 4-hydroxylation) that could be catalyzed by the three cytochrome P450 monooxygenases (Guengerich 2018) encoded by genes that occur in the putative gene clusters in both *T. arundinaceum* and *A. ochraceus*. Similarly, esterification of the butenoyl intermediate to the 8-hydroxyl group could be catalyzed by the acyltransferase encoded by a gene that occurs in both clusters. The ability of the Δ*asp1* mutant to produce the aspinolide analog that lacks the butenoyl substituent but not analogs that have the substituent indicates that the ASP1 PKS catalyzes formation of the diketide precursor of the butenoyl substituent. Finally, the two dehydrogenation reactions in the proposed pathway could be catalyzed by dehydrogenases/oxidoreductases encoded by two genes that occur in both the *T. arundinaceum* and *A. ochraceus* gene clusters. However, it is not clear whether one of these reactions, the reduction of the 4,5-epoxide is catalyzed by an enzyme or occurs spontaneously (Guengerich 2018). Functional analyses of the genes in the putative aspinolide biosynthetic gene cluster combined with biochemical analyses of the resulting mutants should provide information on whether the genes function in aspinolide biosynthesis.

In conclusion, results from a combination of comparative genomic, gene deletion, and transcriptomic analyses in the current study provide evidence that *asp1* and *asp2* are required for aspinolide production and that they are located in a putative aspinolide biosynthetic gene cluster*.* However, additional functional analyses are required to confirm that other genes in the cluster have roles in aspinolide biosynthesis. The ability of both AspC and HA to inhibit fungal growth combined with the retention of antifungal activity of *T. arundinaceum* strains that cannot produce one or the other of these metabolites highlight the complex nature of the antifungal activity of *T. arundinaceum*. This complexity is underscored by the reciprocal increase in aspinolide production in HA-nonproducing mutants and HA production in the aspinolide-nonproducing *asp2* mutant. It remains to be determined whether *T. arundinaceum* strains that are completely blocked in trichothecene and aspinolide production retain any antifungal activity and, if they do, what metabolite(s) is responsible for the activity. Another question raised by the results of the current study is the mechanism(s) that regulates increased HA and aspinolide production in aspinolide-nonproducing and HA-nonproducing *T. arundinaceum* mutants, respectively.

Multiple *Trichoderma* species are used in the biological control of crop diseases caused by other fungi. In some cases, the control has been attributed, at least in part, to the production of antifungal secondary metabolites (Hermosa et al. 2014; Khan et al. 2020). The results of the current study as well as investigations into the genetics and biochemistry of HA production have provided valuable insights into the antifungal activity of *T. arundinaceum* and its potential as a biological control agent.

## Supplementary Information

Below is the link to the electronic supplementary material.

Supplementary file1 (PDF 11921 KB)

## Data Availability

The manuscript has no associated data.

## Declarations

### Disclaimer

Mention of trade names or commercial products in this article is solely for the purpose of providing specific information and does not imply recommendation or endorsement by the US Department of Agriculture. USDA is an equal opportunity provider and employer.
