# Supplementary material for: Identification of polyketide synthase genes required for aspinolide biosynthesis in Trichoderma arundinaceum
Source: Appl Microbiol Biotechnol. 2022 Sep 27;106(21):7153–71. doi: 10.1007/s00253-022-12182-9 (PMC9592644; doi:10.1007/s00253-022-12182-9)
Supplement: Supplementary file 1 — Supplementary file1 (PDF 11921 KB) [file 253_2022_12182_MOESM1_ESM.pdf]

Supplementary Material

Journal: Applied Microbiology and Biotechnology

Identification of polyketide synthase genes required for aspinolide biosynthesis in *Trichoderma arundinaceum*

Rosa E. Cardoza[1 #], Susan P. McCormick[2 #], Inmaculada Izquierdo-Bueno[3 #], Natalia Martínez-Reyes[1], Laura Lindo[1], Daren W. Brown[2], Isidro G. Collado[3], Robert H. Proctor[2]*, Santiago Gutiérrez[1]*

[1]University Group for Research in Engineering and Sustainable Agriculture (GUIIAS), Area of Microbiology, University of León, 24400 Ponferrada, Spain

[2]USDA, Agricultural Research Service, National Center for Agricultural Utilization Research, Mycotoxin Prevention and Applied Microbiology Research Unit, 1815 N University St., Peoria, Illinois, United States 61604

[3]Departamento de Química Orgánica, Facultad de Ciencias, Campus Universitario Río San Pedro s/n, Torre Sur, 4ª planta, Universidad de Cádiz, 11510 Puerto Real, Cádiz, Spain.

Journal:

Applied Microbiology and Biotechnology

* Corresponding authors:

Santiago Gutiérrez (s.gutierrez@unileon.es), phone number: +34 987442060.

Robert H. Proctor (robert.proctor@ars.usda.gov), phone number: +1 309 6816380.

Table S1. Oligonucleotides used in the present work.

**Name**	**Sequence 5´-3´**
**Construction of pΔ*asp1* to delete *asp1* gene.**	
Asp1_5r_F_*Bam*HI (1118 bp)	ggatccGAGCTTTGAAACATTCGG
Asp1_5r_R_*Sma*I	cccgggaatcgcaggtagaaagga
Asp1_3r_F_*Sma*I (1212 bp)	cccgggaagcaggtatctaacgtg
Asp1_3r_R_XhoI	ctcgagcggtactctaacgaggtt
**Construction of pΔ*asp2* to delete *asp2* gene.**	
Asp2_5r_F_*Xba*I (1227 bp)	tctagaggcttgtttgccgcttct
Asp2_5r_R_*Sma*I	cccggggatgagagatgaagagat
Asp2_3r_F_*Sma*I (1142 bp)	cccggggataatggaagagtggta
Asp2_3r_R_*Xho*I	ctcgaggggaacagaagtggaaag
**Amplification of internal regions of TARUN_*asp1* and hph genes**	
Asp1F (1028 bp)	TGTGACTTGCCATCTGGA
Asp1R	GACATTGAGTCTGAGGCA
Db741 (466 bp)	GGATGCCTCCGCTCGAAGTA
Db742	CGTTGCAAGACCTGCCTGAA
**Amplification of 1491 bp and 1410 bp fragments corresponding to the 5´ and 3´ extremes, respectively, of the recombination cassettes designed for *asp1* gene deletion**	
Asp1-5r_rr (1491 bp)	ATCAGATCGCGATCTGAG
TtrpC-d	GTAACCATGCATGGTTGC
PgpdA-d (1410 bp)	ATCATCCACTGCACCTCA
Asp1-3r_rr	GATTCGTACTGTGCAGGA
**Amplification of 1491 bp and 1410 bp fragments corresponding to the 5´ and 3´ extremes, respectively, of the recombination cassettes designed for *asp2* gene deletion**	
Asp2-5r_rr (1603 bp)	AGATCTTGGGTGCCTTTG
Asp2-3r_rr (1338 bp)	CTGCCTTGTTGGCTTTCA

Table S2. tBLASTx analysis using the 23 *Trichoderma arundinaceum* PKSs as query sequences against the *Aspergillus ochraceus* NRRL 35121 genome sequence.

#	Query	Number of HSPs	Lowest E-value	*A. ochraceus* contig #
1	TARUN_493	41	1.10E-8	*A. ochraceus*_contig_0027
2	TARUN_1719	44	1.96E-24	*A. ochraceus*_contig_1086
3	TARUN_2070	17	3.77E-57	*A. ochraceus*_contig_0554
4	TARUN_2474	52	1.72E-12	*A. ochraceus*_contig_1039
5	TARUN_2538	44	3.71E-15	*A. ochraceus*_contig_0571
6	TARUN_2657	24	2.64E-9	*A. ochraceus*_contig_0735
7	TARUN_2893	43	1.97E-63	*A. ochraceus*_contig_0460
8	TARUN_2903	40	5.56E-25	*A. ochraceus*_contig_0508
9	TARUN_2996	25	3.11E-9	*A. ochraceus*_contig_0483
10	TARUN_3319	17	6.84E-49	*A. ochraceus*_contig_0446
11	TARUN_3625	40	5.66E-18	*A. ochraceus*_contig_0027
12	TARUN_3766	43	7.86E-42	*A. ochraceus*_contig_0571
**13**	**TARUN_4144**	**38**	**0.00**	***A. ochraceus*_contig_0382 (*asp1*)**
**14**	**TARUN_4155**	**41**	**1.07E-33**	***A. ochraceus*_contig_0382 (*asp2*)**
15	TARUN_5173	39	1.27E-7	*A. ochraceus*_contig_0728
16	TARUN_5179	27	1.24E-7	*A. ochraceus*_contig_0435
17	TARUN_5779	42	1.89E-24	*A. ochraceus*_contig_0267
18	TARUN_5805	35	1.90E-24	*A. ochraceus*_contig_0571
19	TARUN_6067	47	2.15E-36	*A. ochraceus*_contig_0344
20	TARUN_6263	35	1.25E-7	*A. ochraceus*_contig_0267
21	TARUN_8400	59	3.30E-79	*A. ochraceus*_contig_0055
22	TARUN_9248	56	5.47E-57	*A. ochraceus*_contig_0728
23	TARUN_9303	59	2.22E-11	*A. ochraceus*_contig_0382

HSP: High-Scoring Segment Points.

Table S3. ORFs predicted in the genomic region of *A. ochraceus* containing *asp1, asp2* genes.

Sequence Name (Gene)	Protein (aa)	Predicted function Highest identity	Blastp Score; Accession No.
AOCHR_5353	266	Unknow	$3e^{-54}$; QQE36285.1
AOCHR_5354	289	Aldehyde dehydrogenase	$1e^{-138}$; PLB48697.1
AOCHR_5355	444	P450 monooxygenase	0.0; KXX76482.1
AOCHR_5356	603	P450 monooxygenase	0.0; KXX76483.1
**AOCHR_5357 (*asp2*)**	**2493**	**Pentaketide synthase (PKS)**	**0.0; KXX76491.1**
AOCHR_5358	334	Alpha/beta-hydrolase	$6e^{-145}$; KXX76490.1
AOCHR_5359	539	P450 monooxygenase	0.0; KXX76486.1
AOCHR_5360	246	Short-chain dehydrogenase reductase (SDR)	$1e^{-159}$; XP_001272279.1
AOCHR_5361	587	FAD-linked oxidoreductase	0.0; KXX80981
AOCHR_5362	543	MFS-transporter	0.0; GAQ09988.1
AOCHR_5363	497	Acetyl/acyl transferase	0.0; KXX76481.1
**AOCHR_5364 (*asp1*)**	**2630**	**Diketide synthase (PKS)**	**0.0; KXX76480.1**

Table S4. Spectroscopic data for 3,11-diepiisotrichotriol (**2**) (^{1}H NMR (CD_3OD, 500 MHz), ^{13}C NMR (CD_3OD,125 MHz).

	Proton	δ^{1}H	δ^{13}C	NOESY	HMBC
**1**					
**2**	H-2β	3.66 (d, *J*= 5.3 Hz)	80.7	H-13a, H-4β	C3, C5, C12
**3**	H-3α	4.40 (dd (br), *J* =7.7, 3.6 Hz)	73.9	H-11, H-15, H-4α	C2, C4, C12
**4**	H-4α	2,47 (dd, *J*= 15.2, 7.7 Hz)	39.8	H-4β, H-11, H-3	C2, C5, C12
	H-4β	1.86 (ddd, *J* = 15.2, 5.3, 3.6 Hz)			
**5**	--	-	50.1		
**6**	--	-	41.1		
**7**	H-7a	1,46 (ddt, *J* = 12.6, 5.9, 1.6 Hz)	25.5		
	H-7b	1.94 (m)			C6, C8, C11
**8**	H-8a	1.31 (m)	29.0		C7, C9, C10
	H-8b	2.02 (m)			
**9**	--	-	141.2		
**10**	H-10	5.35 (m)	119.9	H-11, H-16	C6, C8, C11, C16
**11**	H-11α	3.58 (d, *J* = 5.6 Hz)	71.9		C7, C9, C10, C15
**12**	H-12	-	66.6		
**13**	H-13a	2.99 (d, *J* = 4.1 Hz)	47.9	H-13b, H-2	C2, C5, C12
	H-13b	2.80 (d, *J* = 4.1 Hz)		H-13a, H-7, H-14	C2, C5, C12
**14**	H-14β	0.77 (s)	6.4	H-7, H-13	C5, C6, C12
**15**	H-15α	0.87 (s)	16.1	H-7α, H-8α, H-11, H-3	C5, C6, C7, C11
**16**	H-16	1.69 (dq, *J* = 1.4, 0.8 Hz)	23.3		C8, C9, C10

Table S5. Quantification of ergosterol and squalene production by Ta37 (control), and the Δ*asp1 and* Δ*asp2* mutants, in samples from 48 h cultures grown in PDB medium

	Dry weight (g)	Squalene (mgS/g DW)*	Ergosterol (mgE/g DW)**
Ta37 48h	0.103 ± 0.002	1.349[a] ± 0.131	56.372[a] ± 2.759
Δ*asp1* 48h	0.101 ± 0.001	1.312[a] ± 0.256	47.988[a] ± 7.831
Δ*asp2* 48h	0.100 ± 0.002	1.328[a] ± 0.107	51.43 [a] ± 8.562

n= 2, ANOVA.

[a] On each column, values followed by same superscript letters are not significantly different ($p < 0.05$).

* mg S/g DW.- milligrams squalene / gram dry weight.

** mg E/g DW.- milligrams ergosterol / gram dry weight.

Table S6. Percentages of radial growth inhibition (RI) of *R. solani* by Ta37, ∆*asp1*, and ∆*asp2* grown on cellophane membranes, after 7 and 10 days of incubation after the placement of the pathogen on the plates.

	**7 days**	**10 days**
**Ta37**	72.01[a] ± 2.06	24.64[a] ± 0.74
**∆*asp1***	70.21[a] ± 0.54	25.13[a] ± 3.50
**∆*asp2***	86.91[b] ± 4.05	53.59[b] ± 14.88

n= 3, ANOVA.

[a,b] On each column, values followed by different superscript letters are significantly different ($p < 0.05$). n=3.

Table S7. ORFs predicted in the genomic region of *T. arundinaceum* containing aspinolide cluster and flanking genes.

Gene	Protein (aa)	Predicted function Highest identity	Blastp Score; Accession No.
TARUN_4134	113	Unknown	$2e^{-60}$; KAF3074171.1
TARUN_4135	350	Aminoglycoside phosphotransferase	0.0; OPB46131.1
TARUN_4136	285	Integral membrane protein	$3e^{-156}$; OPB46130.1
TARUN_4137	213	NAD(P)-dehydrogenase	$3e^{-123}$; KAF3074450.1
TARUN_4138	568	Unknown	0.0; UKZ52441.1
TARUN_4139	336	FAD-dependent oxidoreductase	0.0; KAH6603977.1
TARUN_4140	921	P450 monooxygenase	0.0; KAF3074454.2
TARUN_4141	1112	ABC transporter	0.0; OPB46124.1
TARUN_4142	509	Cytochrome P450 monooxygenase	0.0; KAH6603980.1
TARUN_4143	1757	Non-ribosomal peptide synthetase	0.0; PTB75339.1
**TARUN_4144 (*asp1*)**	**2521**	**Diketide synthase (PKS)**	**0.0; KXX76480.1**
TARUN_4145	500	Acetyl/acyl transferase	0.0; KXX76481.1
TARUN_4146	422	P450 monooxygenase	0.0; KXX76482.1
TARUN_4147	612	P450 monooxygenase	0.0; KXX76483.1
TARUN_4148	240	Unknown	$3e^{-152}$; PKK53437.1
TARUN_4149	506	FAD-dependent monooxygenase	0.0; KXX76484.1
TARUN_4150	236	Short chain dehydrogenase-reductase (SDR)	$1e^{-126}$; PKK53440.1
TARUN_4151	586	FAD-linked oxidoreductase	0.0; KXX80981
TARUN_4152	349	Unknown	$2e^{-175}$; OPB46113.1
TARUN_4153	513	P450 monooxygenase	$8e^{-150}$; XP_022490940
TARUN_4154	306	Alpha/beta-hydrolase	$1e^{-122}$; PMD39540.1
**TARUN_4155 (*asp2*)**	**2338**	**Pentaketide synthase (PKS)**	**0.0; XP_007584097.1**
TARUN_4156	520	Zn2Cys6 transcription factor	0.0; OPB46110.1
TARUN_4157	308	NAD(P)-binding protein	$6e^{-165}$; KAH8125998.1
TARUN_4158	515	P450 monooxygenase	0.0; XP_040774906.1
TARUN_4159	680	ATP-binding transmembrane protein	2e-76; KAI1455363.1
TARUN_4160	538	HET-domain-containing protein	$1e^{-59}$; KAH86569742.1
TARUN_4161	445	Unknown	0.0; KAH6603990.1
TARUN_4162	320	Oxidoreductase	0.0; KAH6603991.1
TARUN_4163	425	Oxidoreductase	0.0; CP_024745537.1
TARUN_4164	1560	Unknown	0.0; UKZ69085.1
TARUN_4165	308	Oxidoreductase	0.0; QYS9915.1

Note that genes in the aspinolide biosynthetic cluster have been shaded in yellow.

Table S8. RNAseq analysis of expression of genes located at the aspinolide cluster genomic region in 48h PDB grown mycelia from Ta37 (wild-type strain) and Δtri6.66 mutant.

Gene	TPM Ta37*	TPM Δtri6.66*	nTPM Ta37**	nTPM Δtri6.66**
TARUN_4134	1	11.9	0.003	0.036
TARUN_4135	0.83	2.99	0.002	0.009
TARUN_4136	1	1	0.003	0.003
TARUN_4137	6.84	8.62	0.018	0.027
TARUN_4138	5.13	27.24	0.013	0.085
TARUN_4139	3.47	1.56	0.009	0.005
TARUN_4140	0.32	0.28	0.001	0.001
TARUN_4141	2.08	5.61	0.005	0.017
TARUN_4142	1	2.06	0.03	0.006
TARUN_4143	1.49	2.39	0.004	0.007
**TARUN_4144 (*asp1)***	**1,711.73**	**2,714.15**	**4.427**	**8.435**
**TARUN_4145**	**1,724.71**	**2,975.72**	**4.461**	**9.247**
**TARUN_4146**	**8,057.28**	**12,939.50**	**20.839**	**40.211**
**TARUN_4147**	**3,129.12**	**3,976.48**	**8.093**	**12.357**
**TARUN_4148**	**480.87**	**609.78**	**1.244**	**1.895**
**TARUN_4149**	**1,377.12**	**2,072.25**	**3.562**	**6.440**
**TARUN_4150**	**19,369.32**	**23,140.92**	**50.097**	**71.913**
**TARUN_4151**	**8,642.59**	**8,657.61**	**22.353**	**26.905**
**TARUN_4152**	**111.9**	**114.98**	**0.289**	**0.357**
**TARUN_4153**	**16,023.81**	**27,024.08**	**41.444**	**83.980**
**TARUN_4154**	**3,856.28**	**4,542.59**	**9.974**	**14.117**
**TARUN_4155 (*asp2*)**	**1,155.02**	**1,974.77**	**2.987**	**6.137**
TARUN_4156	**5.04**	5.55	0.013	0.017
TARUN_4157	1.89	2.55	0.005	0.008
TARUN_4158	83.19	118.66	0.215	0.369
TARUN_4159	0.86	3.47	0.002	0.011
TARUN_4160	0.54	1.95	0.001	0.006
TARUN_4161	3.27	5.89	0.008	0.018
TARUN_4162	2.73	1	0.007	0.003
TARUN_4163	18.51	2.47	0.048	0.008
TARUN_4164	118.26	189.14	0.306	0.588
TARUN_4165	9.46	9.37	0.024	0.029
TARUN_5502 (actin)	386.64	321.79		

*TPM: Transcripts detected for each gene per million of total reads

**nTPM: TPM of each gene normalized versus TPM of actin (housekeeping) of each sample

Note that data corresponding to genes putatively assigned to the aspinolides biosynthetic cluster are written in bold type.

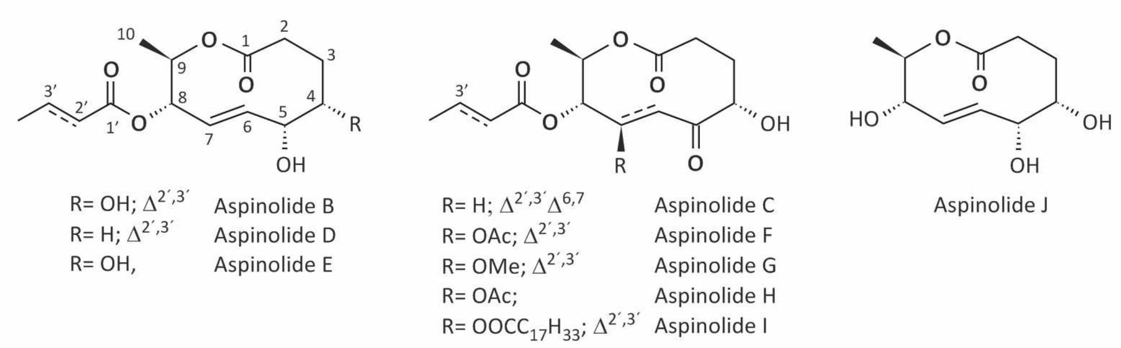

Figure S1. Chemical structure of aspinolides produced by *Trichoderma arundinaceum* (Izquierdo-Bueno et al., 2018; Malmierca et al., 2015).

Izquierdo-Bueno, I., Moraga, J., Cardoza, R.E., Lindo, L., Hanson, J.R., Gutiérrez, S., Collado, I.G. 2018. Relevance of the deletion of the *Tatri4* gene in the secondary metabolome of *Trichoderma arundinaceum*. *Org. Biomol. Chem.* 16: 2955. doi: 10.1039/cBob00338f.

Malmierca, M.G., Barua, J., McCormick, S.P., Izquierdo-Bueno, I., Cardoza, R.E., Alexander, N.J., Hermosa, R., Collado, I.G., Monte, E., Gutiérrez, S. 2015. Novel aspinolide production by *Trichoderma arundinaceum* with a potential role in *Botrytis cinerea* antagonistic activity and plant defence priming. *Environmental Microbiology*. 17: 1103-1118.

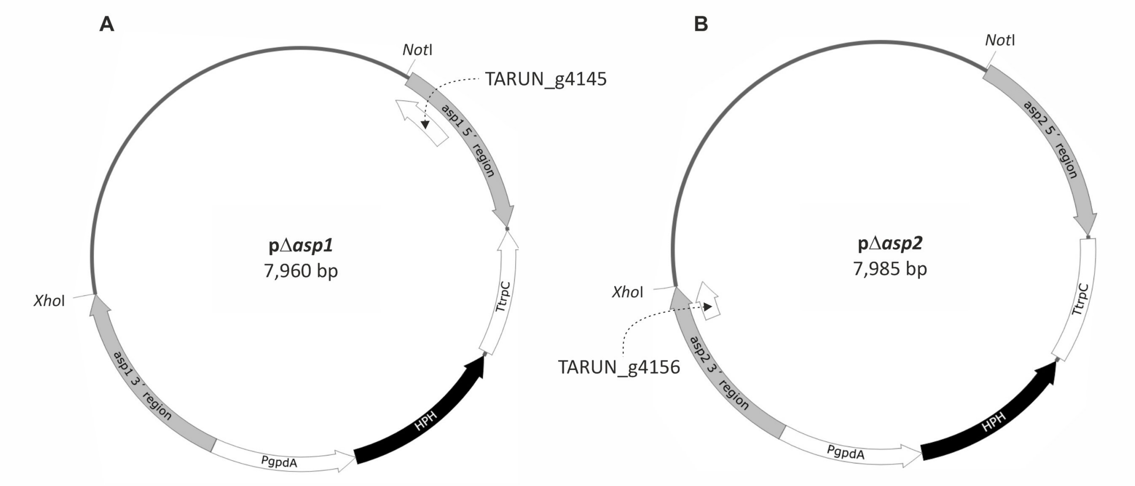

Figure S2. **A**. *asp1* deletion plasmid pΔ*asp1*. asp1-5´-region and asp1-3´-region correspond to the 1,117-bp 5´ and 1,218-bp 3´ flanking regions, respectively, of the *T. arundinaceum asp1* coding region. **B**. *asp2* deletion plasmid pΔ*asp2* (7,985 bp). asp2-5´-region and asp2-3´-region correspond to the 1,227-bp 5´ and 1,142-bp 3´ flanking regions, respectively, of the *T. arundinaceum asp2* coding region. PgpdA is the promoter region of the glyceralhedyde-3-phosphate dehydrogenase gene from *Aspergillus nidulans*; *hph* is the *E. coli* hygromycin resistance gene; and TtrpC is the *A. nidulans trpC* terminator. The 5´ region of the TARUN_4145 gene that is included in the asp1-5´-region fragment is also indicated. Similarly, the 5´ region of the TARUN_4156 gene included in the asp2-3´-region fragment is indicated.

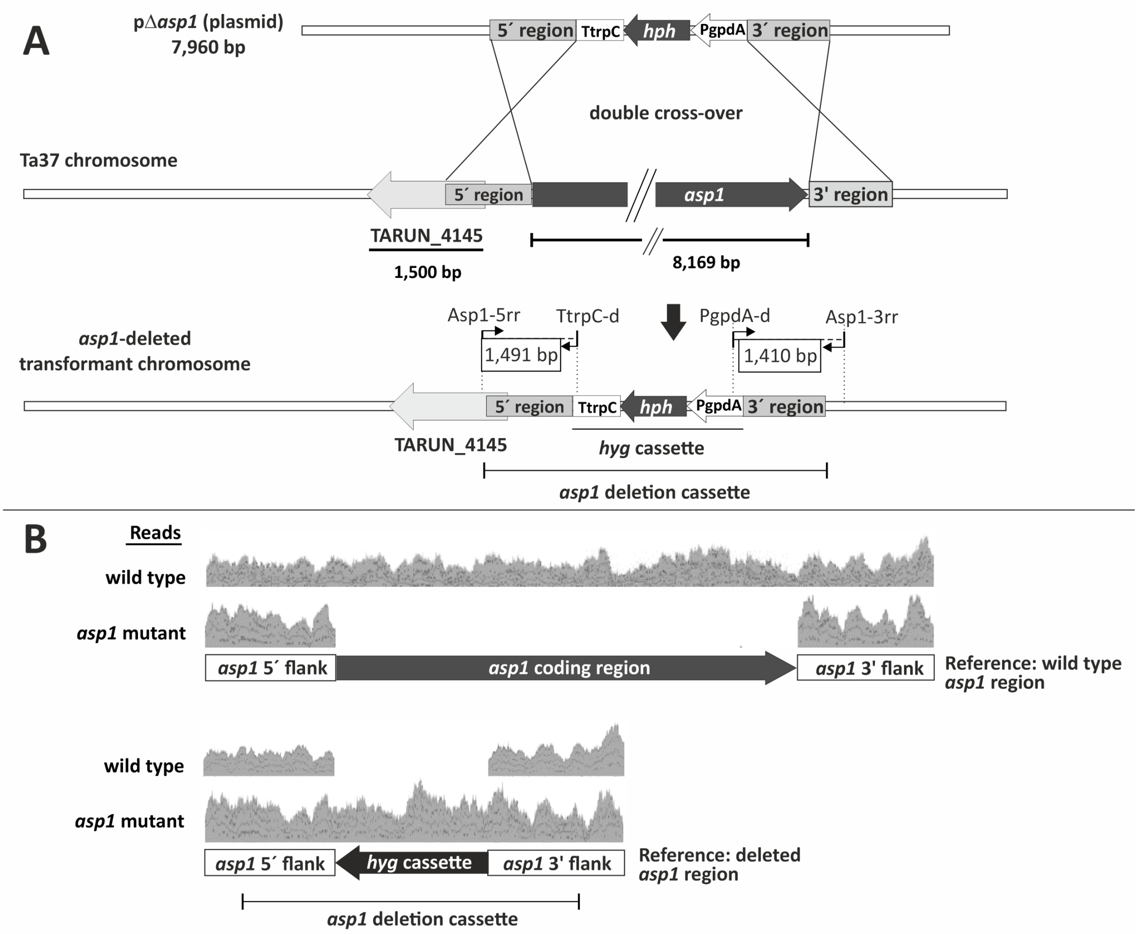

Figure S3. *asp1* gene deletion. **A.** double cross-over event with plasmid pΔ*asp1* (**Figure S2A**). The position of the diagnostic PCR fragments 1,410 bp and 1,391 bp to determine whether the *asp1* deletion cassette integrated into the *asp1* region via homologous recombination in the *asp1*-5´ and *asp1*-3´ flanking regions, respectively, are indicated in the boxes. **B**. *In silico* sequence read mapping was used to confirm the PCR results for *asp1* mutant strain Δasp1.9. For read mapping experiments, whole genome sequences of the wild-type strain Ta37 and the *asp1* mutant were generated with an Illumina MiSeq instrument. After processing, the resulting reads were subject to the *Map Reads to Reference* analysis in CLC Genomics Workbench 20.0. The reference sequences used in the analysis were the wild-type *asp1* region (14,339 bases) and the predicted sequence resulting from deletion of *asp1* using the *asp1* deletion cassette (9,456 bases). The sequences in the *asp1* 5' and 3' flanks were the same in both reference sequences.

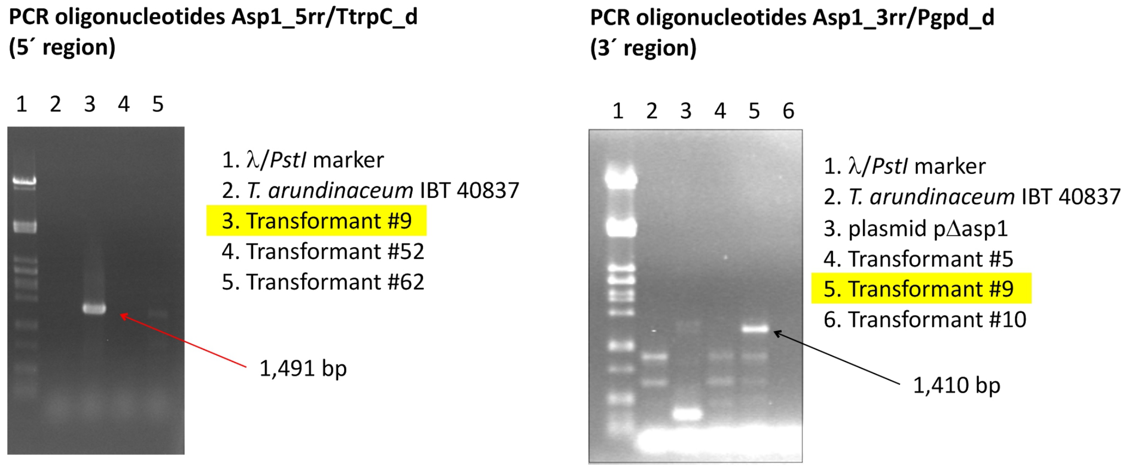

Figure S4. PCR analysis of selected *T. arundinaceum* transfomants obtained with plasmid pΔ*asp1* using primer pairs Asp1_5rr/TtrpC_d (left panel), and Asp1_3rr/Pgpd_d (right panel) that amplify 1,491 bp and 1,410 DNA fragments corresponding to the 5´ and 3´ extremes of the recombination cassette designed for *asp1* deletion. λ/*PstI* = DNA from phage lambda digested with *PstI*, used as molecular size marker. Transformant #9 is highlighted in yellow as PCR generated the expected DNA fragments indicating replacement of *ASP1* with *hph*.

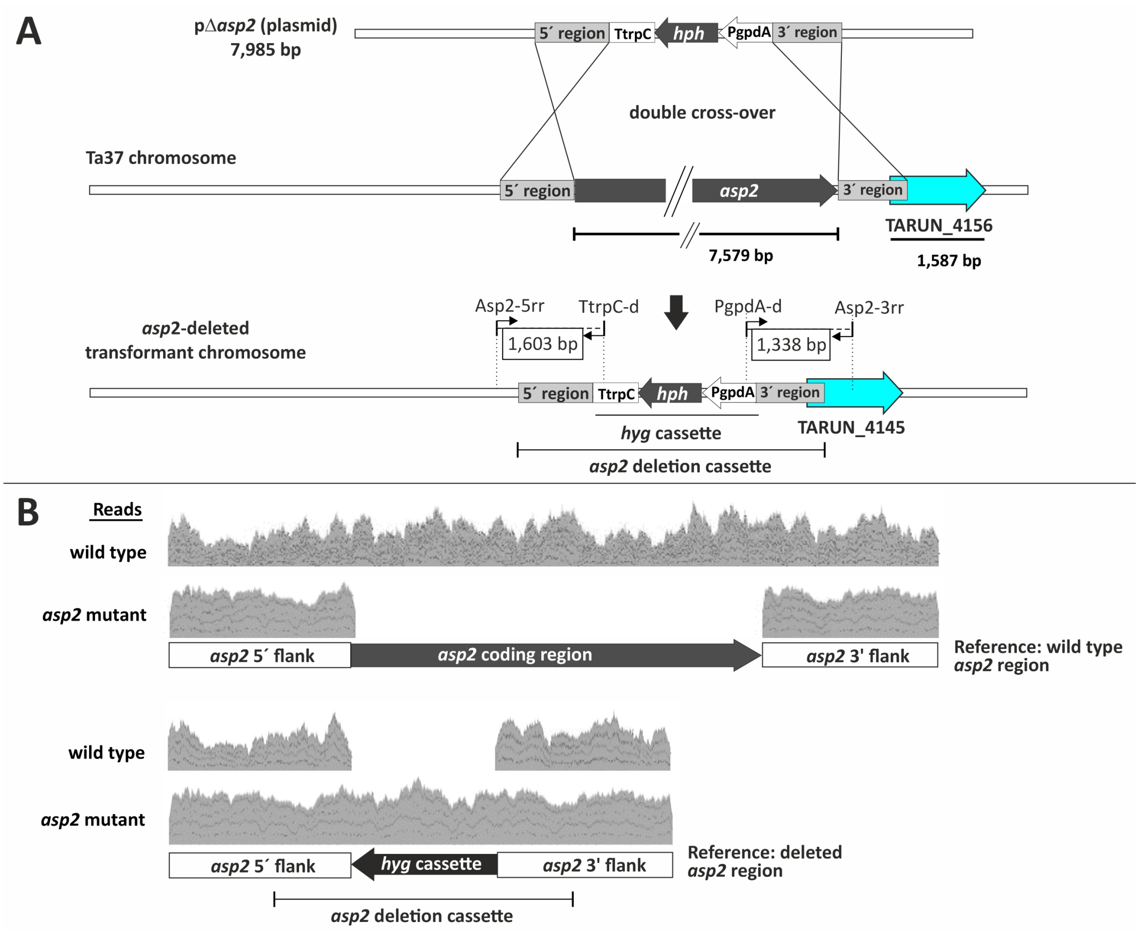

Figure S5. Experiments for detection of *asp2* gene deletion. **A.** double cross-over event with plasmid pΔ*asp2* (**Fig. S2B**). The position of the diagnostic PCR fragments 1,603 bp and 1,338 bp to determine whether the *asp2* deletion cassette integrated into the *asp2* region via homologous recombination in the *asp2*-5´ and *asp2*-3´ flanking regions, respectively, are indicated in the boxes. **B**. *In silico* sequence read mapping was used to confirm the PCR results for *asp2* mutant strain Δasp2.3. For read mapping experiments, whole genome sequences of the wild-type strain Ta37 and the *asp2* mutant were generated with an Illumina MiSeq instrument. After processing, the resulting reads were subject to the *Map Reads to Reference* analysis in CLC Genomics Workbench 20.0. The reference sequences used in the analysis were the wild-type *asp2* region and the predicted sequence resulting from deletion of *asp2* using the *asp2* deletion cassette. The sequences in the *asp2*-5’ and 3’ flanks were the same in both reference sequences.

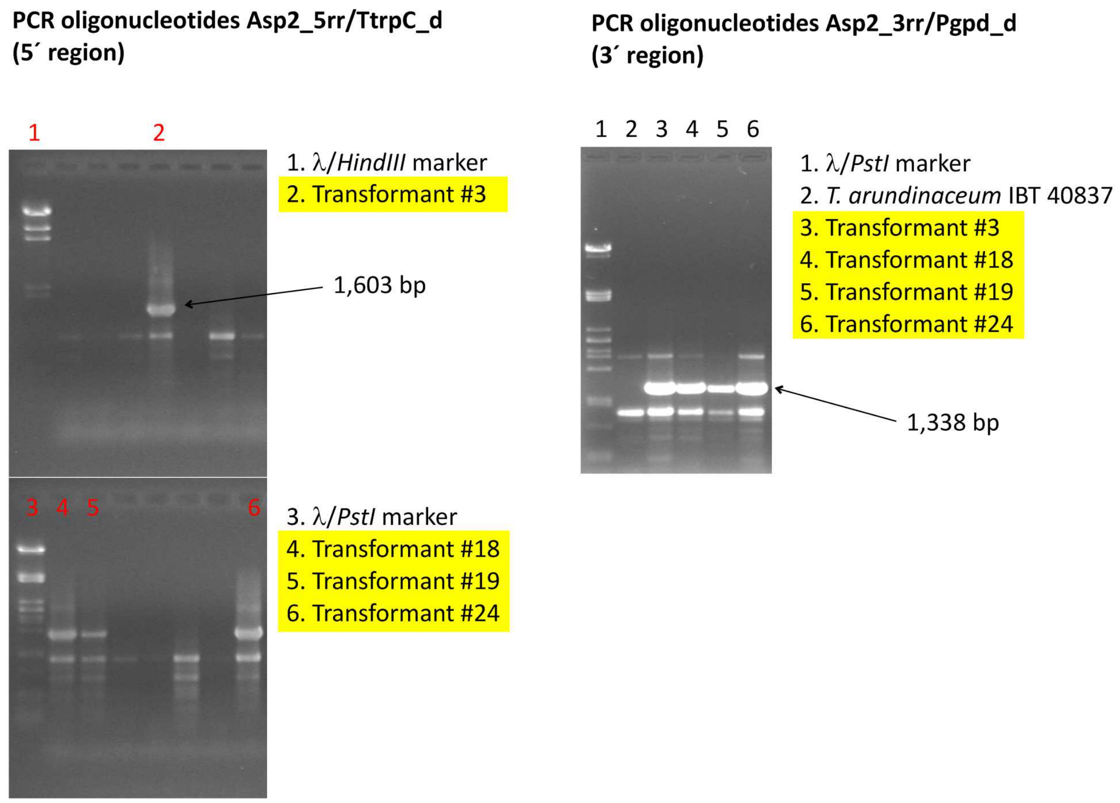

Figure S6. PCR analysis of selected *T. arundinaceum* transfomants obtained with plasmid pΔ*asp2* using primer pairs Asp2_5rr/TtrpC_d (left panel), and Asp2_3rr/Pgpd_d (right panel) designed to amplify 1,603 bp and 1,338 bp fragments corresponding to the 5´ and 3´ extremes of the recombination cassette designed for *asp2* deletion. λ/*HindIII*= DNA from phage lambda digested with *HindIII*, used as molecular size marker. Transformants highlighted in yellow generated the expected DNA fragments indicating replacement of *asp2* with *hph*.

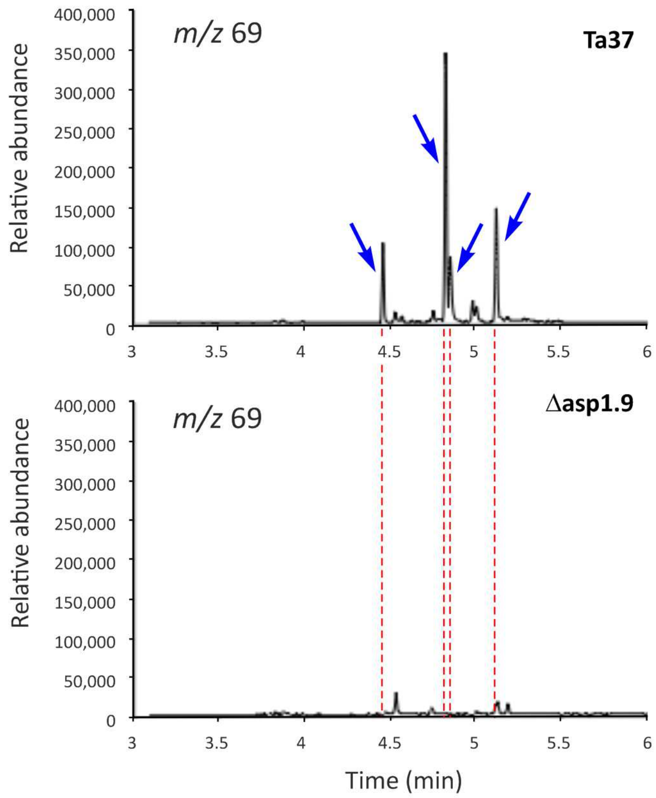

Figure S7. Gas chromatography-mass spectrometry analysis of aspinolide production by wild-type *T. arundinaceum* strain IBT 40837 (**upper panel**) and *asp1* mutant strain Δasp1.9 (**lower panel**) using the fragment ion with m/z 69. Blue arrows indicate the peaks corresponding detected aspinolide signals.

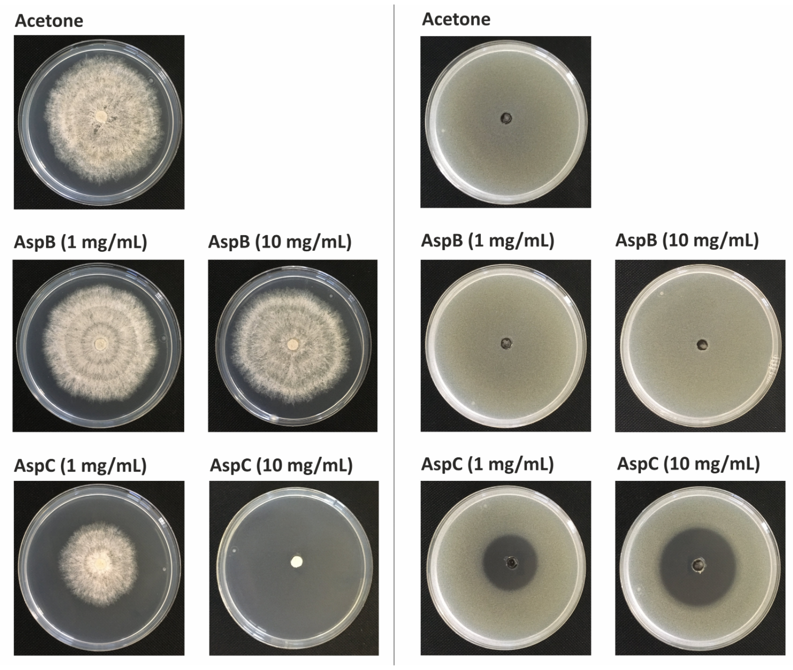

Figure S8. Antifungal assay of pure AspB and AspC against *Rhizoctonia solani* R43 (left panel) and *Kluyveromyces marxianus* CECT 1018 (right panel).

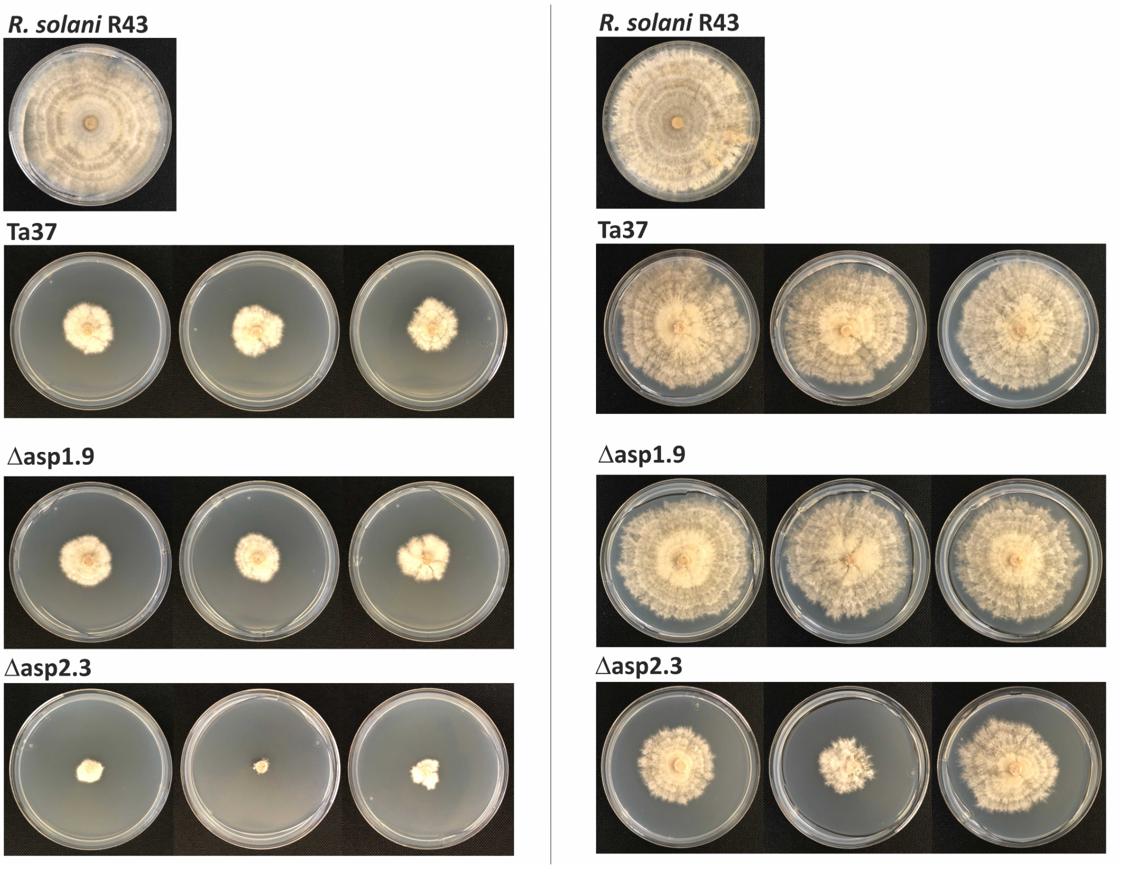

Figure S9. Antifungal activity of *T. arundinaceum* strains Ta37 (wild type), Δasp1.9, and Δasp2.3 against the fungal phytopathogen *Rhizoctonia solani* (strain R43) in a cellophane membrane assay. Plates were incubated for 7 days (left panel) or 10 days (right panel). Percentages of radial growth inhibition are shown in **Table S6**.

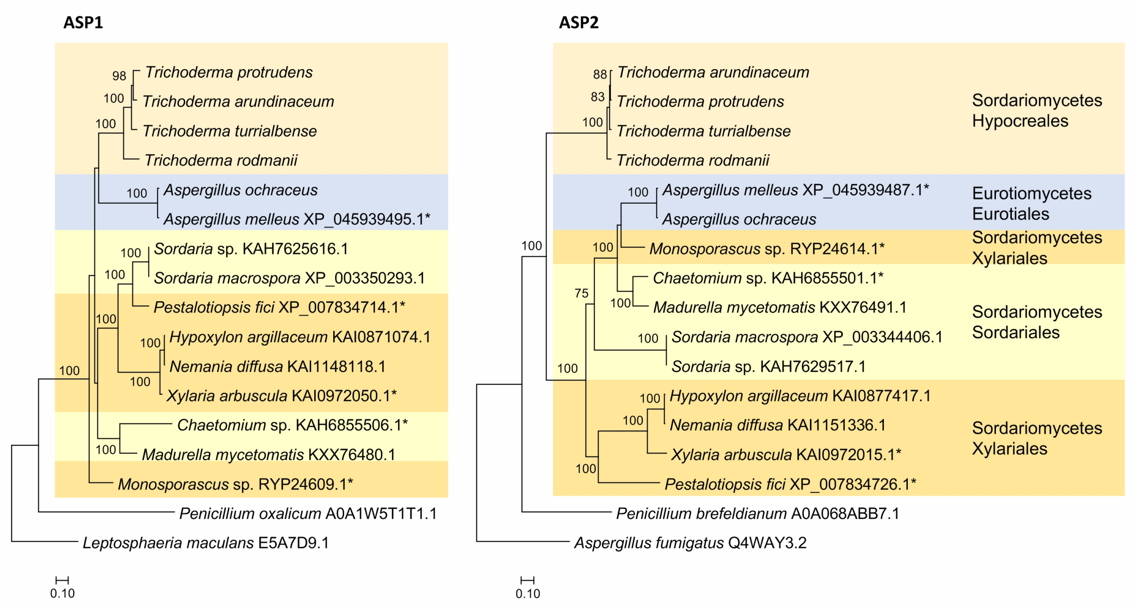

Figure S10. Maximum likelihood trees inferred from predicted amino acid sequences of the ASP1 and ASP2 homologs from *A. ochraceus*, *Trichoderma* species and 10 other fungi identified by BLASTx analysis. The trees were inferred using the program IQ-Tree (version 1.6.7). Numbers near branches are bootstrap values determined from 1,000 pseudoreplicates. Because they are not considered significant, bootstrap values of less than 70 are not shown. Sequences identified by BLASTx analysis indicated by the GenBank accession number following the fungal name. An asterisk after the accession number indicates that the *asp1* and *asp2* homologs occur on the same contig in the genome sequence of the corresponding fungus. The colored blocks indicate the class (e.g., Sordaryomycetes) and order (e.g., Hypocreales) of the fungi with ASP1 and ASP2 homologs. The ASP1-like sequences from *Leptosphaeria maculans* and *Penicillium oxalicum* and the ASP2-like sequences from *Aspergillus fumigatus* and *Penicillium brefeldianum* were selected as outgroups from among the 10 best hits in BLASTp analysis using the *T. arundinaceum* ASP1 and ASP2 sequences as queries against NCBI's UniProtKB/Swiss-Prot(swissprot) database. The selection of the outgroups was based on preliminary maximum likelihood trees of ASP1 and ASP2 that included the protein sequence obtained from BLASTp analyses of the UniProtKB/Swiss-Prot(swissprot) database.
